# Avidity-optimized TCR-T cells target KRAS neoantigens for potent cancer clearance and tumor microenvironment remodeling

**DOI:** 10.3389/fimmu.2026.1736294

**Published:** 2026-01-26

**Authors:** Zhaoduan Liang, Fengqiong Guan, Bingling Wu, Wenfang Chen, Ye Tian, Wenxuan Cai, Yi Li

**Affiliations:** 1 Bioland Laboratory, Guangzhou, Guangdong, China; 2 Guangzhou Regenerative Medicine and Health GuangDong Laboratory, Guangzhou, Guangdong, China; 3 T-cell Immunity Optimized Cure (TIOC) Therapeutics Limited, Zhongshan, Guangdong, China; 4 State Key Laboratory of Respiratory Disease, Guangzhou Institutes of Biomedicine and Health, Chinese Academy of Sciences, Guangzhou, Guangdong, China

**Keywords:** cancer, KRAS, neoantigen, optimized avidity, TCR-T cells, tumor microenvironment

## Abstract

**Introduction:**

Neoantigens from the Kirsten rat sarcoma viral oncogene homolog (KRAS) are specific cancer therapeutic targets. However, to date, no immune product targeting KRAS neoantigens has been approved for clinical use, and key challenges regarding efficacy and generalizability remain.

**Methods:**

In this study, we isolated a natural human T-cell antigen receptor (TCR) 0 that specifically recognized human leukocyte antigen (HLA)-A*11:01+ T2 cells pulsed with KRAS G12V_8–16_ peptides. However, TCR0 gene-transduced T cells demonstrated inadequate response to tumor cell lines. We generated T cells expressing a TCR0 mutant, being designated as TCR3.

**Results:**

TCR3-T cells showed significantly optimized avidity and response to tumor cell lines, retained specificity for the KRAS G12V8–16 peptide with no response to normal cells, killed tumor cells that highly expressed programmed cell death-ligand 1 *in vitro* and *in vivo*, proliferated without being seriously affected by indoleamine 2,3-dioxygenase, resisted transforming growth factor β, and infiltrated and recruited other immune cells to the tumor site through chemokines.

**Discussion:**

TCR3 may be useful for KRAS neoantigen-targeted clinical immunotherapy, help resolve cancer immune escape, and enhance clinical effectiveness and safety.

## 1 Introduction

Antibody- and adoptive cell-based immunotherapies overcome the limitations of conventional therapies for cancer and elicit exciting, durable responses in cancer patients (1–4). Approved cancer immunotherapies commonly target tumor-associated antigens (TAAs) or cancer-testis antigens that are also expressed in normal cells or immune-privileged sites, such as glycoprotein 100 (gp100) (1), melanoma antigen gene-A4 (MAGE-A4) (2), human epidermal growth factor receptor-2 (3), cluster of differentiation 19 (CD19) (4), and B-cell maturation antigen (4). Owing to this weak tumor specificity, these kinds of targets not only limit the dosage of drugs, but also harbor risks of on-target off-tumor toxicities, which is a key issue to be addressed. Profiting from next-generation sequencing-based technology and *in-silico* analysis, many somatic mutations within tumors have been identified, including nonsense, missense, site splicing, frameshift, and rearrangements (5). Pathogenic mutations are important for tumor formation and progression and can be present on the surface of cancer cells as peptides, that serve as neoantigens and can be used by the immune system to distinguish cancer cells from normal cells (5–7).

The Kirsten rat sarcoma viral oncogene homolog (KRAS) is a small guanosine triphosphatase (GTPase) that cycles between inactive and active forms. guanosine triphosphate (GTP)-bound KRAS is activated and transmitted through multiple cellular pathways to trigger cell growth, division, and differentiation. GTPase-activating proteins that hydrolyze GTP can convert KRAS into an inactive guanosine diphosphate (GDP)-bound state, terminating the physiological cycle of cells (8, 9). Mutations in KRAS impair GTP hydrolysis, locking it in an active GTP-bound state, causing durative activation of downstream pathways and leading to tumorigenesis, thus being identified as a driver oncogene (10). Approximately 14% of all cancers carry mutated KRAS, particularly higher in pancreas adenocarcinoma (90%), colorectal carcinoma (50%), and lung adenocarcinoma (32%) (9, 11). Over 80% of KRAS missense mutations occur in glycine at the 12^th^ site (G12), which is substituted with aspartic acid (D), valine (V), cysteine (C), and arginine (R), resulting in G12D, G12V, G12C, and G12R variants, respectively (12). A series of mutated KRAS epitopes have been confirmed to be present in human leukocyte antigen (HLA)-I molecules on the surface of tumor cells, such as the HLA-A*11:01 restricted KRAS G12V_8–16_ peptide, which has been validated as an immunological target (6, 7, 9, 11). Such consistently high-frequency tumor-driven KRAS mutants have been identified as attractive therapeutic neoantigens.

Recently, AMG510 (Sotorasib) and MRTX849 (Adagrasib) inhibitors have been approved for patients with KRAS G12C mutation. These antagonists have promising clinical anti-tumor activities, although drug resistance generally emerges after treatment (10, 13). Vaccines against immunogenic mutated KRAS elicit specific T-cell immunity, but fail to show clinical benefits (11). The infusion of tumor-infiltrating lymphocytes or T-cell antigen receptor (TCR)-T cells recognizing HLA-C*08:02-restricted KRAS G12D_10–18_ or KRAS G12D_10–19_ peptide can mediate significant cancer regression. However, one patient showed progressive disease, which results from the loss of HLA-C*08:02 (14, 15). Preclinically, TCR (ImmTAC) or TCR-mimetic-antibody bispecific T cell engager shows *in vitro* T-cell activation and cytotoxicity of tumor cells (7, 9). The *in vivo* efficacy of a murine TCR only inhibits the growth of tumor and extends animal life in a xenograft model (8). Mutated KRAS upregulates the expression of programmed cell death-ligand 1 (PD-L1) and CD47, and inhibits the infiltration of CD8^+^ T cells, resulting in the suppression of both innate and adaptive immune surveillance (16–18). The KRAS-enhanced immunosuppressive tumor microenvironment (TME) may account for the poor efficacy of immunotherapies targeting mutated KRAS.

In this study, we aimed to optimize T-cell immunity for the treatment of KRAS mutant-related cancers with high-avidity TCRs. The TCRs specifically recognized the KRAS neoantigen (HLA-A*11:01 restricted KRAS G12V_8–16_ antigen), but not the wild-type KRAS, and conferred the transduced T cells with efficient tumor killing *in vitro* and *in vivo*, with no response to healthy cells. The T cells demonstrated tumor infiltration and resistance of immune suppression in the TME, promoting the recruitment of immune cells to the tumor. These TCR molecules will provide improved efficacy in the clinical treatment of patients with KRAS mutations.

## 2 Materials and methods

### 2.1 Cell lines and PBMCs

Using lentiviral transduction, we constructed the following cell lines to express HLA-A*11:01 or human PD-L1 from their parental cells: SW480-A1101 (KRAS G12V^+^/HLA-A*11:01^+^, human colon cancer), CFPAC-1-A1101 (KRAS G12V^+^/HLA-A*11:01^+^, human pancreatic ductal adenocarcinoma), SHP-77-A1101 (KRAS G12V^+^/HLA-A*11:01^+^, human lung carcinoma), T2-A1101 (KRAS G12V^-^/HLA-A*11:01^+^, human lymphoblast), and SW480-A1101-hPD-L1. HCC-366 (KRAS G12V^-^/HLA-A*11:01^+^, human lung carcinoma) and SW620 (KRAS G12V^+^/HLA-A*11:01^-^, human colon adenocarcinoma) were not transduced. All cell lines (**Supplementary Table 1**) were grown in medium supplemented with 10% fetal bovine serum (Sigma, cat# F8687) at 37°C in a humidified 5% CO_2_ incubator for less than one month, Mycoplasma negative tested with a polymerase chain reaction (PCR) kit (Beyotime, China, cat# C0301S), and authenticated with Short Tandem Repeat (STR) from supplier. Peripheral blood mononuclear cells (PBMCs) were purchased from Milestone^®^ Biotechnologies (China), which had been approved by the Human Subject Research Ethics Committee of the Bioland Laboratory (No. GDL-IRB2022-007).

### 2.2 Normal cells

Thirteen types of normal cells (**Supplementary Table 2**) were used as target cells, ten of which were engineered to express HLA-A*11:01. Cells were cultured in supplemented medium at 37°C in a humidified 5% CO_2_ incubator for up to two weeks. Mycoplasma testing and STR authentication were performed, confirming all lines were mycoplasma-negative and properly identified.

### 2.3 Isolation of KRAS G12V_8-16_-HLA-A*11:01 specific CD8^+^ T-cell clones

On day 1, PBMCs were stained with KRAS G12V_8-16_–HLA-A*11:01 tetramer and anti-CD8 antibody, and specific CD8^+^ T cells were sorted by a fluorescence-activated cell sorting (FACS) (BD Bioscience). Sorted cells were co-cultured with feeder cells in TexMACS™ Medium (Miltenyi Biotec, cat# 130-097-196) supplemented with 5% human AB serum (GeminiBio, cat# 100-512), KRAS G12V_8-16_ peptide, and cytokines (IL-2 (10 IU/mL), IL-7 (10 ng/mL), IL-21 (30 ng/mL)) for 14 days. On day 15, a second sort was performed, then positive CD8^+^ T cells were diluted to single-cell level and expanded into clones with feeder-cell stimulation for 14 days. On day 29, the clones which grew well were selected to analyze the positive rate (KRAS G12V_8-16_–HLA-A*11:01 tetramer) then sorted. Medium was replaced every 3–4 days. TCR genes from selected clones were identified by 5′-RACE (Clontech, cat# 634860) and sequenced (Sangon Biotech).

### 2.4 Generation of avidity-optimized TCR mutants

Mutations were introduced into the CDR1 region of the TCR α chain by overlap PCR using primers listed in **Supplementary Table 3**. Products were cloned into a phagemid vector, electroporated into TG1 *E. coli* (Lucigen, cat# 60502-1), and used to generate a TCR phage display library. Variants with enhanced affinity were isolated using the KRAS G12V_8-16_–HLA-A*11:01 complex. Clones showing higher ELISA signals were sequenced, and optimized TCR binding was validated by functional cell assays.

### 2.5 Preparation of TCR-T cells

Hybrid TCR genes containing variable regions fused to murine constant regions were cloned into a lentiviral vector. Lentiviral particles were produced by co-transfected HEK-293T cells (Cobioer) with helper vectors. On day 1, CD8^+^ and CD4^+^ T cells were isolated from PBMCs using EasySep™ selection kits (STEMCELL, cat# 17853 & 17852) and activated with Dynabeads^®^ Human T-Activator CD3/CD28 (Gibco, cat# 11132D). On day 2, activated T cells were transduced with lentivirus for 48 h. On day 4, transduced cells were expanded for 9 days. On day 13, TCR expression (**Supplementary Table 4**) was analyzed by flow cytometry (CytoFLEX S, Beckman Coulter), after which TCR-T cells were cryopreserved. Cells were maintained in ImmunoCult™-XF T Cell Expansion Medium (STEMCELL, cat# 10981) with IL-2 (100 IU/mL). Unless specified, TCR-T cells refer to TCR-expressing CD8^+^ T cells.

### 2.6 ELISPOT assay

TCR-T cells and peptide-loaded T2-A1101 cells were co-cultured on ELISPOT plates pre-coated with anti-IFN-γ antibody (BD Biosciences, cat# 551849) for 16 h at 37°C under 5% CO_2_. After cell lysis, biotinylated anti-IFN-γ antibody and streptavidin-HRP conjugate were sequentially incubated. Spots were developed with 3-amino-9-ethylcarbazole substrate (BD Biosciences, cat# 551951) and counted using an AID iSpot Reader Spectrum (AID GmbH).

### 2.7 Lactate dehydrogenase releasable assay

Supernatants contained releasable LDH were incubated with CytoTox 96^®^ reagent (Promega, cat# G1780) for 30 min, followed by addition of stop solution. Absorbance was measured at 490 nm (Multiskan SkyHigh, Thermo Scientific). Cytotoxicity was calculated as per the manufacturer’s instructions.

### 2.8 Alanine scanning assay

Each amino acid in the parental peptide was substituted with alanine (alanine residues were changed to glycine; see **Supplementary Table 5**). TCR-T cells were co-cultured with peptide (10^-10^ M)-loaded T2-A1101 cells at an effector-to-target (E:T) ratio of 5:1. Cytotoxicity measured by LDH release was used to evaluate alanine-scanning results.

### 2.9 Chemokine and cytokine assays

Chemokines and cytokines were measured using LEGENDplex™ HU Proinflam Chemokine Panel 1 (BioLegend, cat# 741081) and LEGENDplex™ HU Th1 Panel (BioLegend, cat# 741035). Diluted supernatants and standards were incubated with capture beads for 2 h, followed by detection antibodies (1 h) and SA-PE (30 min). Fluorescence was acquired by flow cytometry, and concentrations were calculated according to the manufacturer’s protocols.

### 2.10 TCR-T-cell proliferation assay

TCR-T cells pre-stained with CellTrace™ Violet (Invitrogen, cat# C34557) were co-cultured with irradiated target cells for 5 days, in the presence or absence of a 1:1 mixture of 3-hydroxyanthranilic acid (KKL Med, cat# KM16148) and kynurenine (InvivoChem, cat# V13205) (KHAA), or recombinant human TGF-β1 (rhTGF-β1; R&D Systems, cat# 7754-BH-005/CF). KHAA was dissolved in DMSO to 0.4 M stock and tested at 50–400 μM; DMSO concentration was matched in all groups. RhTGF-β1 was tested at 0.1, 1, and 10 ng/mL. ImmunoCult™-XF T Cell Expansion Medium was used throughout. Proliferation was assessed by flow-cytometric detection of diluted CellTrace™ Violet signal.

### 2.11 Transwell assay for monitoring cell movements

TCR-T cells, T-cell deleted PBMCs, and CellTrace™ Violet-labeled CD3^+^ T cells were placed in the upper chamber of a transwell plate (Corning, cat# 3415), with target cells in the lower chamber. After 3 days in ImmunoCult™-XF T Cell Expansion Medium, infiltration and recruitment of TCR-T cells, CD3^+^ T cells, dendritic cells, macrophages, and monocytes were analyzed by antibody staining and flow cytometry (**Supplementary Table 4**).

### 2.12 Treatment with TGF-β1

TCR-T cells were co-cultured with irradiated target cells at ratios of 1:5 or 1:10, with or without rhTGF-β1 (0.1, 1, or 10 ng/mL), for 5 days in ImmunoCult™-XF T Cell Expansion Medium. Cells were fixed, permeabilized, and stained for Foxp3 (**Supplementary Table 4**); Foxp3^+^ frequency was determined by flow cytometry.

### 2.13 Mouse xenograft models

All animal procedures were approved by the Experimental Animal Ethics Committee of Bioland Laboratory (No. 2022-0018). Female NOD/SCID-IL2rγ^-^/^-^ (B-NDG) mice (Biocytogen), aged 6–9 weeks, were housed under SPF conditions with sterile food and water. Mice were subcutaneously injected on the dorsum with SW480-A1101 (2×10^6^), CFPAC-1-A1101 (1.3×10^6^), SHP-77-A1101 (3×10^6^), or SW480-A1101-hPD-L1 (2×10^6^) cells. When tumor volume reached 30–70 mm³, tumor-bearing mice (n = 20 per model) were randomized into four groups using Microsoft Excel and received a single intravenous injection of PBS, CD8^+^ T cells (3×10^7^), TCR0-T cells (3×10^7^), or TCR3-T cells (3×10^7^). Tumors were measured twice weekly with calipers; volume was calculated as (length × width²)/2 (19). Mice were anesthetized with isoflurane (REWARD, cat# R510-22) and euthanized by CO_2_ inhalation when average tumor volume in a group reached 1000 mm³.

### 2.14 Statistical analysis

Data were analyzed and visualized using GraphPad Prism v8.0. Results are presented as mean ± SEM. Differences between groups were assessed by unpaired two-tailed Student’s t-test; *P* < 0.05 was considered significant. Significance levels: *P < 0.05, **P < 0.01, ***P < 0.001, ****P < 0.0001; ns (not significant) *P* > 0.05. Brackets indicate compared groups. Each experiment was repeated at least three times. Independent experiments used T cells from the same healthy donor. Sample sizes are provided in figure legends.

## 3 Results

### 3.1 Isolation of wild-type TCR and optimization of the avidity

**Figure 1:**
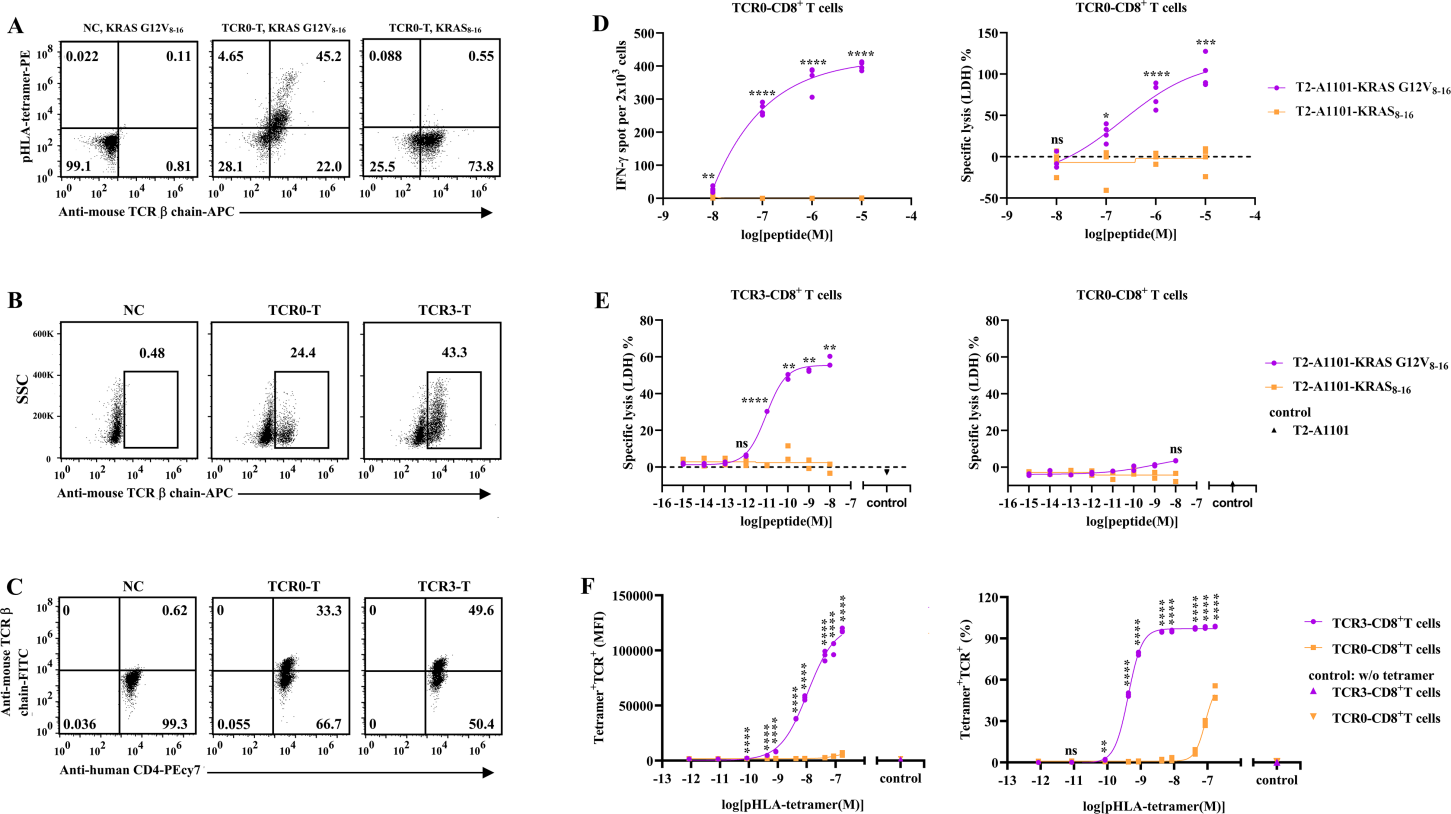
Comparison of functions between wild-type and optimized TCRs. **(A)** The expression and recognition of TCR0 (wild type) in CD8^+^ T cells. **(B)** The expressions of TCR0 and TCR3 (mutation) in CD8^+^ T cells. **(C)** The expressions of TCR0 and TCR3 in CD4^+^ T cells. The isolated TCR0 and optimized TCR3 were transduced into purified CD8^+^ T or CD4^+^ T cells, and the expression and recognition of TCR0 or TCR3 was respectively tested by anti-mouse TCRβ chain antibody and pHLA-tetramer. **(D)** The IFN-γ secretion and cytotoxicity of TCR0-CD8^+^ T cells. TCR0-CD8^+^T cells were co-cultured with T2-A1101 cells, which were loaded with a series of concentrations of KRAS G12V_8-16_-peptide or KRAS_8-16_-peptide, at E:T ratio of 1:10 (IFN-γ) or 5:1 (cytotoxicity) for 16 h. **(E)** Cytotoxicity of TCR3- and TCR0-CD8^+^ T cells. The effect cells were respectively co-cultured with T2-A1101 cells, which were pulsed with a series of concentrations of KRAS G12V_8-16_-peptide or KRAS_8-16_-peptide, at E:T ratio of 5:1 for 16 h. The IFN-γ secretion was detected by enzyme-linked immunospot (ELISPOT) assay, and the supernatant was analyzed by LDH assay. **(F)** The binding to pHLA-tetramer of TCR3- and TCR0-CD8^+^ T cells. TCR3- and TCR0-CD8^+^ T cells were respectively incubated with a series of concentrations of the tetramer of KRAS G12V_8-16_-HLA-A*11:01 complex, then the fluorescence signal of tetramer was detected by flow cytometer. Error bars indicated the SEM (n=2-4).

We isolated a wild−type TCR, designated TCR0, from a healthy donor using the KRAS G12V_8-16_–HLA−A11:01 complex (**Supplementary Figure 1**). TCR0 utilizes the variable−region genes TRAV12−2 and TRBV7−9. Hybrid genes combining the TCR0 variable region with the murine constant region were transduced into polyclonal CD8^+^ T cells, resulting in TCR0 expression on >70% of cells in the resulting TCR0−CD8^+^ T−cell population (**Figure 1A**). The majority of TCR0−CD8^+^ T cells bound the KRAS G12V_8-16_–HLA−A11:01 tetramer but not the wild−type KRAS_8-16_–HLA−A*11:01 tetramer (**Figure 1A**). Upon recognition of T2−A1101 cells pulsed with KRAS G12V_8-16_ peptide (10^-5^ M), TCR0−CD8^+^ T cells produced significant IFN−γ and lysed target cells, whereas no response was observed against cells pulsed with the wild−type KRAS_8-16_ peptide (10^-5^ M) (**Figure 1D**). These data confirm that TCR0 specifically recognizes the KRAS G12V_8-16_ peptide. However, when the peptide concentration was reduced to 10^-8^ M, TCR0−CD8^+^ T−cell activity became undetectable (**Figure 1D**). The functional avidity of TCR0 on TCR−T cells, calculated using a previously reported formula, was 2.168 × 10^-7^ M (**Figure 1D**) (20, 21). Following bio−panning of a TCR0−CDR1α mutant phage library (**Supplementary Figure 2A**), a variant termed TCR3 was isolated (**Supplementary Figures 2B, C**). TCR3 carries five amino−acid substitutions in the CDR1α region (data not shown) and showed higher expression in both CD8^+^ and CD4^+^ T cells compared with the parental TCR0 (**Figures 1B, C**). TCR3−CD8^+^ T cells exhibited strong and specific lysis of T2−A1101 cells pulsed with KRAS G12V_8-16_ peptide even at a concentration as low as 10^-^¹¹ M (**Figure 1E**). The functional avidity of TCR3 was determined to be 9.211 × 10^-^¹² M, representing a 23,537−fold improvement over TCR0. In contrast, TCR0−CD8^+^ T cells showed no detectable cytotoxicity under the same conditions (**Figure 1E**). Furthermore, both the mean fluorescence intensity and the percentage of TCR3−T cells binding to the KRAS G12V_8-16_–HLA−A*11:01 tetramer were dose−dependent and significantly higher than those of TCR0−T cells across all tetramer concentrations tested (**Supplementary Figure 3**). The EC_50_ values decreased by 12.7−fold and 203−fold, respectively (**Figure 1F**). Collectively, these results demonstrate that the engineered TCR3 retains original specificity while exhibiting substantially enhanced functional and binding avidity compared with the parental TCR0.

### 3.2 TCR3-T cells exhibited enhanced antigen-specific cytotoxicity *in vitro*

**Figure 2:**
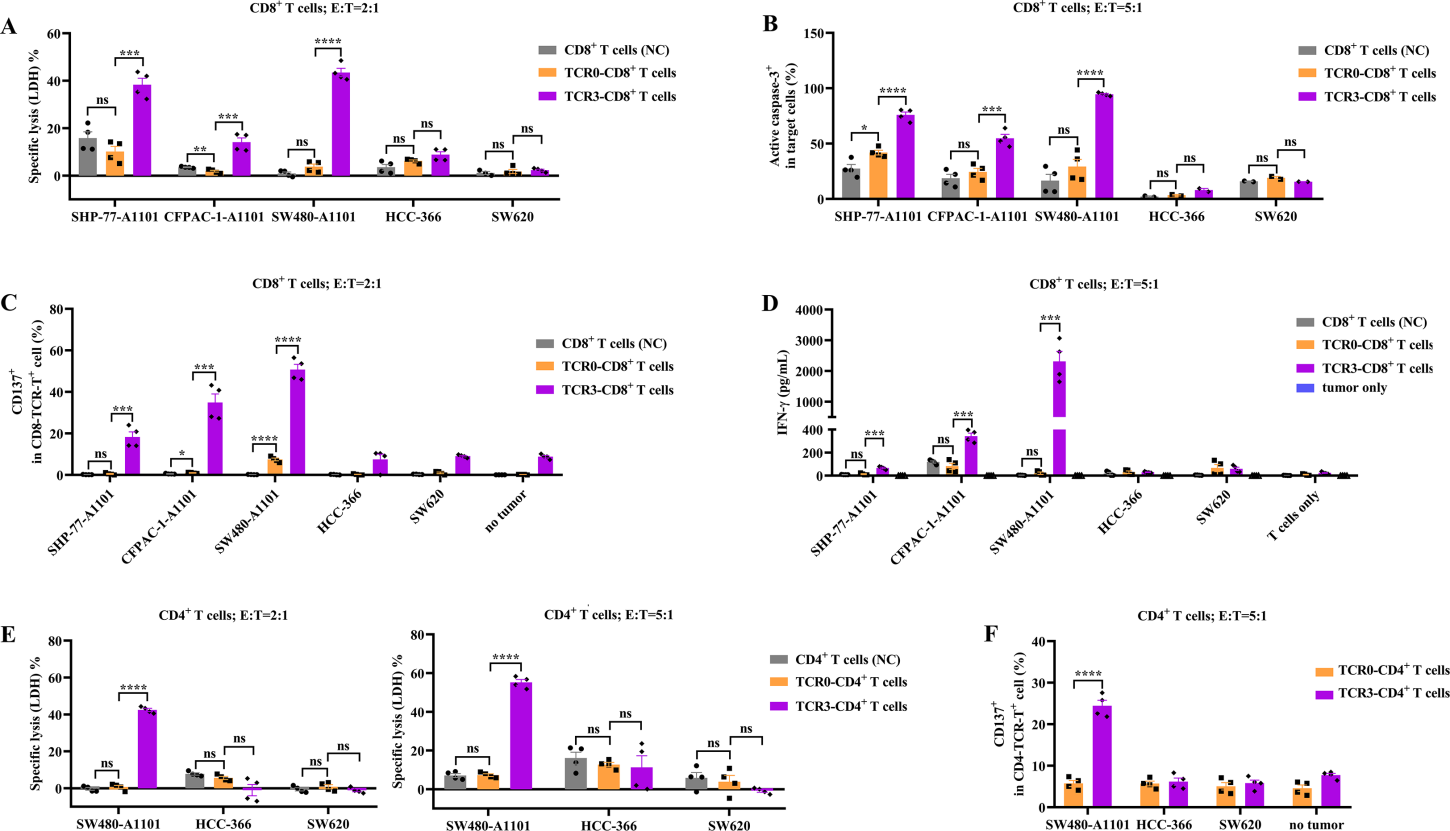
Comparison of *in vitro* cytotoxicity and T-cells activation between wild-type and optimized TCRs. The released LDH **(A)** and active caspase-3 **(B)** between TCR0- and TCR3-CD8^+^ T cells. The CD137 expression **(C)** and IFN-γ secretion **(D)** between TCR0- and TCR3-CD8^+^ T cells. The released LDH **(E)** and CD137 expression **(F)** between TCR0- and TCR3-CD4^+^ T cells. Expanded CD8^+^ or CD4^+^ T cells were used as negative control (NC) of TCR0- and TCR3-T cells. NC, TCR0-T cells and TCR3-T cells were respectively co-cultured with target cells in the E:T ratio of 5:1 or 2:1 for 16 h, and the cytotoxicity represented by LDH and active caspase-3 and T-cells activation represented by CD137 expression and IFN-γ secretion were detected and comparison. Error bars indicated the SEM (n=4).

To assess the tumor-killing capacity of high-avidity TCR3-T cells, we compared TCR3 and TCR0 activity against KRAS G12V^+^/HLA-A*11:01^+^ tumor cells (SHP-77-A1101, CFPAC-1-A1101, and SW480-A1101), as well as control cells lacking either the KRAS mutation (HCC-366) or HLA-A*11:01 (SW620). TCR3-CD8^+^ T cells exhibited superior cytotoxicity. Compared to TCR0-CD8^+^ T cells, TCR3-CD8^+^ T cells demonstrated significantly elevated target cell lysis (LDH release, **Figure 2A**); apoptosis induction (active caspase-3; **Figure 2B**; **Supplementary Figure 4**); T cell activation (CD137 expression; **Figure 2C**; **Supplementary Figure 5**); IFN-γ secretion (**Figure 2D**; **Supplementary Figure 6**). Notably, both TCR3- and TCR0-CD8^+^ T cells showed minimal activity against control cells (HCC-366 and SW620), confirming antigen specificity (**Figures 2A–D**). Untransduced CD8^+^ T cells exhibited negligible cytotoxicity or activation. TCR3 enabled CD4^+^ T cell-mediated killing. TCR3-CD4^+^ T cells effectively lysed SW480-A1101 cells (**Figure 2E**) and expressed CD137 (**Figure 2F**, **Supplementary Figure 7**), while showing no response to antigen-negative controls. In contrast, TCR0-CD4^+^ T cells lacked cytotoxic or activation capacity (**Figures 2E, F**). These findings demonstrated that the higher avidity of TCR3-T cells conferred potent, antigen-restricted tumor targeting *in vitro*, with functional advantages over TCR0 in both CD8^+^ and CD4^+^ T cell subsets.

### 3.3 TCR3-T cells could eliminate tumors *in vivo*

**Figure 3:**
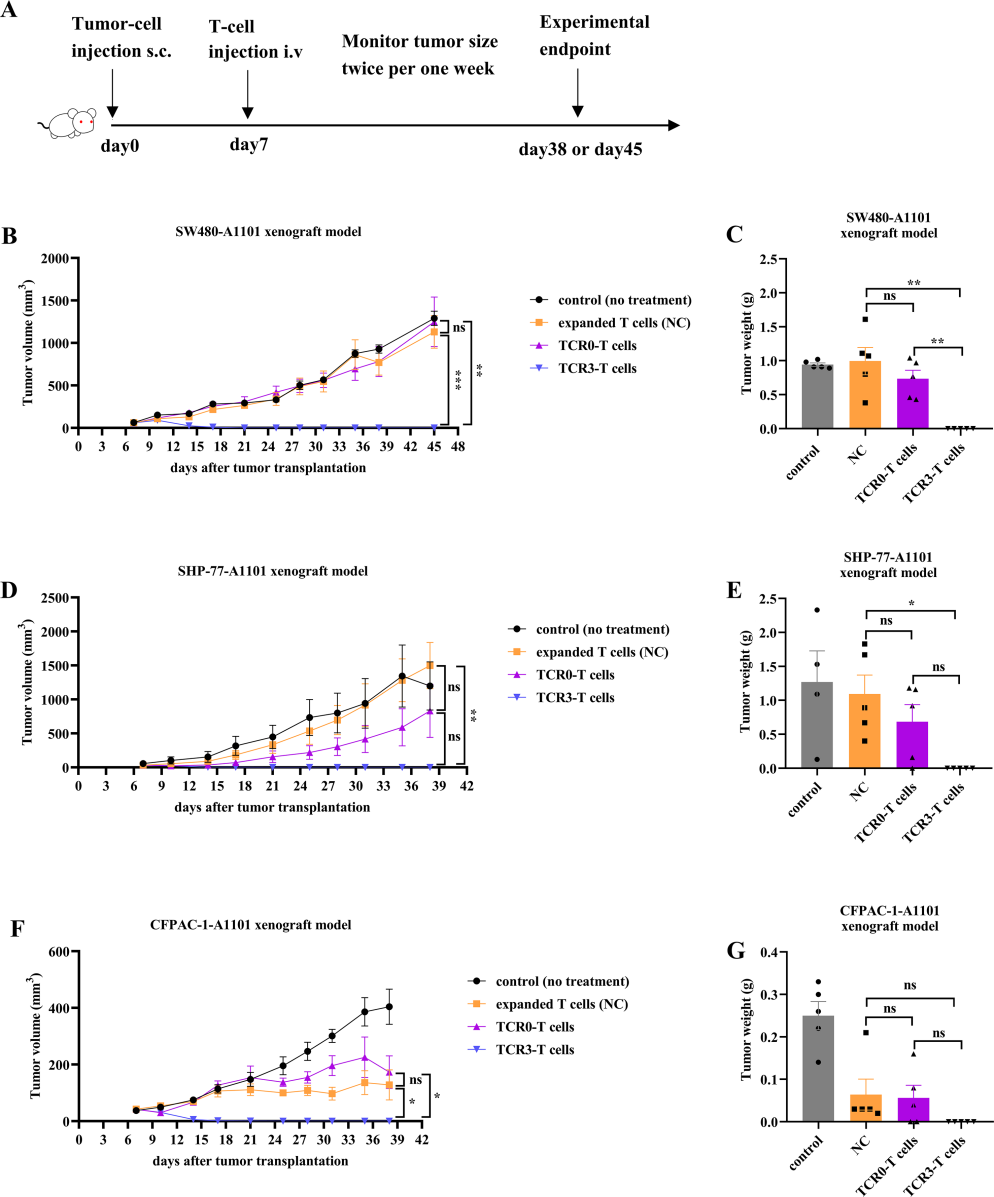
Comparison of *in vivo* anti-tumor capacity between wild-type and optimized TCRs. **(A)** The schematic illustration of the experimental design. Manipulations were initiated for all cohorts (number of mice=20) on day 7. The cohorts included control, expanded T cells, TCR0-T cells and TCR3-T cells. The curves of tumor average volume and time for SW480-A1101 xenograft tumor **(B)**, SHP-77-A1101 xenograft tumor **(D)** and CFPAC-1-A1101 xenograft tumor **(F)** administrated with tested articles for 45 days or 38 days. The average tumor weight for SW480-A1101 xenograft tumor **(C)**, SHP-77-A1101 xenograft tumor **(E)**, and CFPAC-1-A1101 xenograft tumor **(G)** administrated with tested articles at experimental endpoint. Error bars indicated the SEM (n=5).

To evaluate the antitumor efficacy of TCR3-T cells *in vivo*, we established subcutaneous xenograft models in NOD/SCID-IL2rγ^−/−^ (B-NDG) mice using three A1101-positive tumor lines: intestinal cancer (SW480-A1101), lung cancer (SHP-77-A1101), and pancreatic cancer (CFPAC-1-A1101). Upon tumor formation, mice received a single intravenous infusion of TCR3-T cells, TCR0-T cells, or expanded T cells, with tumor growth monitored biweekly (**Figure 3A**). Without treatment, three control xenograft tumors continuously grew and reached endpoint (**Figures 3B–G**). Compared with the group of control, expanded T cells showed variable inhibition across models. TCR0-T cells outperformed expanded T cells in most models (e.g., 44.50% volume inhibition for SHP-77-A1101) but exhibited inconsistent efficacy (e.g., −35.03% volume inhibition for CFPAC-1-A1101). In contrast, TCR3-T cells mediated sustained tumor suppression, achieving complete regression without rhIL-2 support in all models (**Figures 3B–G**). Their efficacy significantly surpassed both TCR0-T and expanded T cells (*P* < 0.05), demonstrating that high-avidity TCR3-T cells enabled durable tumor elimination *in vivo*.

### 3.4 TCR3-T cells overcame PD-L1-mediated immune resistance

**Figure 4:**
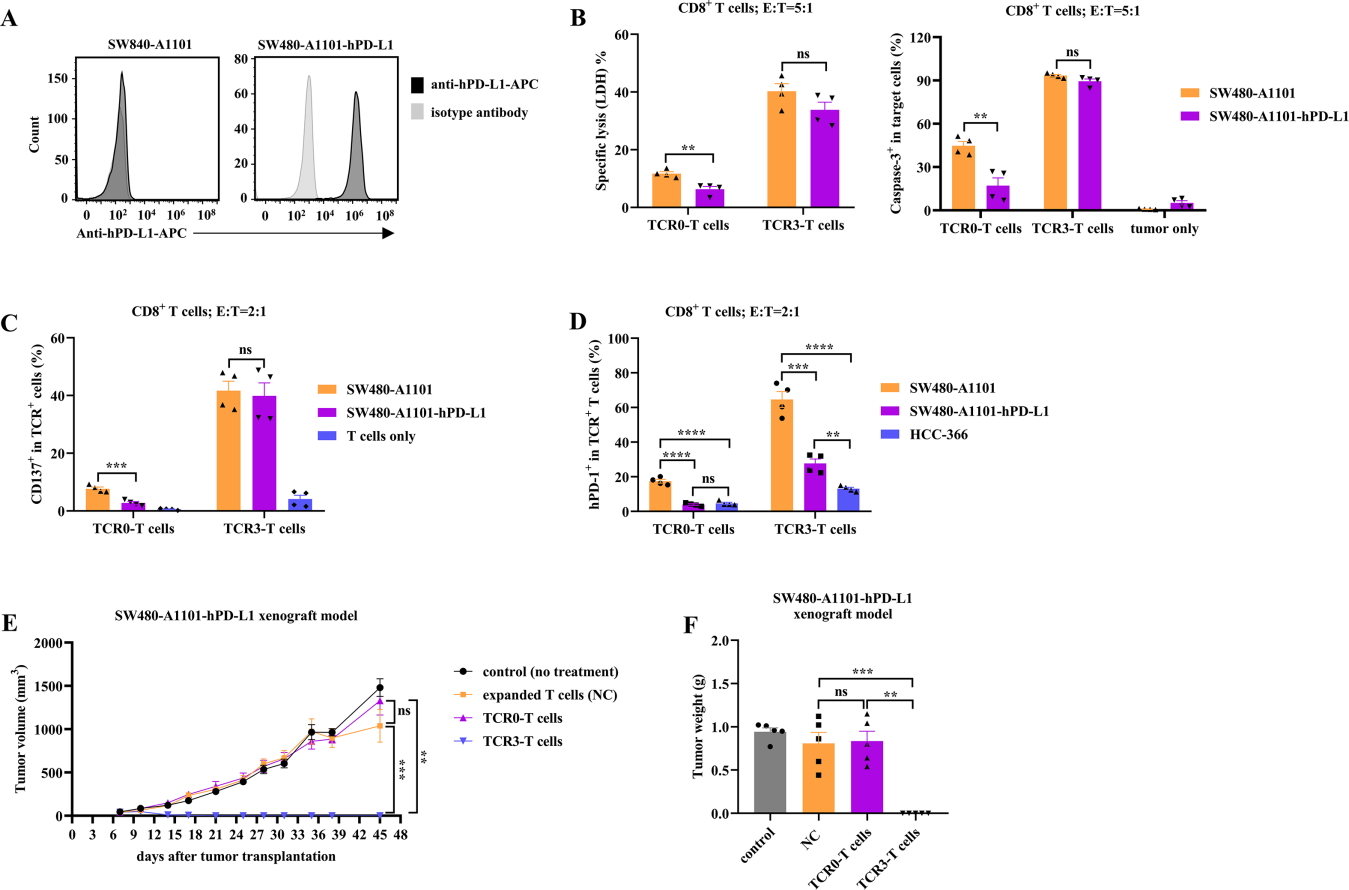
Comparison of the effect of hPD-L1 on wild-type and optimized TCRs. **(A)** FACS histogram plots demonstrating the expression of hPD-L1 between SW480-A1101 and SW480-A1101-hPD-L1 cells. **(B)** The released LDH and active caspase-3 between TCR0- and TCR3-T cells. **(C)** The CD137 expression between TCR0- and TCR3-T cells. **(D)** The PD-1 expression between TCR0- and TCR3-T cells. TCR0- and TCR3-T cells were respectively co-cultured with SW480-A1101, SW480-A1101-hPD-L1 and HCC-366 cells at the E:T ratio of 5:1 or 2:1 for 16 h, then the released LDH, active caspase-3, CD137 and hPD-1 were tested. The effect of TCR0- and TCR3-T cells on *in vivo* tumor growth of SW480-A1101-hPD-L1 xenograft model. The experimental design was the same as **Figure 3A**, and the results were shown as the average tumor volume **(E)** and weight **(F)**. Error bars indicated the SEM (n=4-5).

To evaluate whether TCR3-T cells could counteract PD-L1-mediated immunosuppression, we engineered SW480-A1101-hPD-L1 cells, which exhibited high PD-L1 expression compared to parental SW480-A1101 cells (**Figure 4A**). TCR3-T cells retained cytotoxicity despite high PD-L1. TCR0-T cells showed significantly reduced killing capacity against SW480-A1101-hPD-L1, while TCR3-T cells retained robust cytotoxicity with only marginal inhibition (**Figures 4B, C**; **Supplementary Figures 8**, **9**). Programmed cell death-1 (PD-1) was modulated by TCR3-T cells. Upon recognizing SW480-A1101, TCR3-T cells upregulated PD-1, but this was attenuated against SW480-A1101-hPD-L1, suggesting PD-L1-induced feedback inhibition (**Figure 4D**, **Supplementary Figure 10**). In contrast, TCR0-T cells exhibited minimal PD-1 induction, further highlighting TCR3’s superior activation strength. Despite high PD-L1 expression, TCR3-T cells achieved complete elimination of SW480-A1101-hPD-L1 xenografts, whereas TCR0-T cells failed to control tumor growth (**Figures 4E, F**). These findings demonstrated that high-avidity TCR3-T cells not only enhanced tumor targeting but also counteracted PD-L1-mediated immunosuppression, supporting its potential as a robust immunotherapeutic strategy.

### 3.5 TCR3-T cells exhibited resistance to IDO-mediated immunosuppression

**Figure 5:**
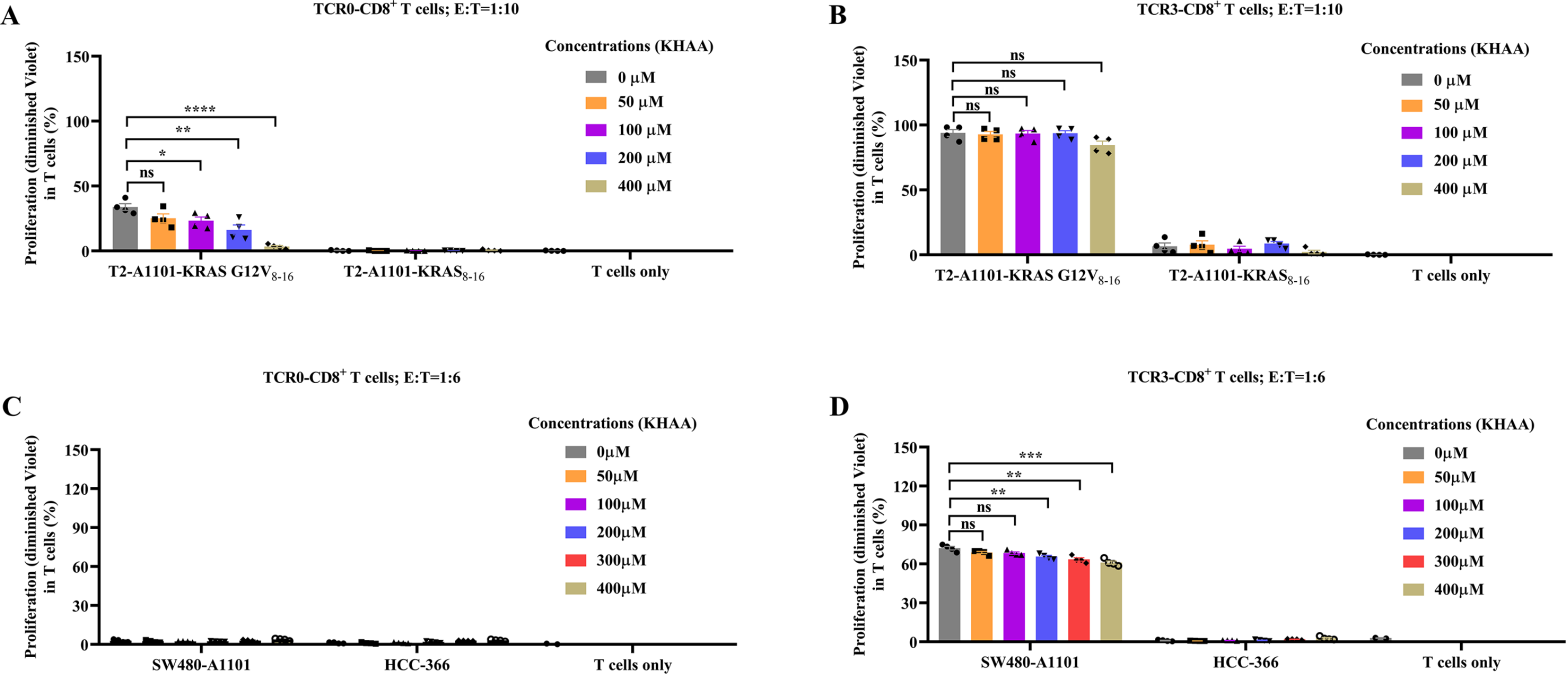
Effect of kynurenine and 3-HAA mixture (KHAA) on the proliferation of wild-type and optimized TCRs. After co-cultured with peptide (5E-6M)-pulsed T2-A1101 cells, the proliferation of TCR0-T cells **(A)** and TCR3-T cells **(B)** in the absence or presence of KHAA. After co-cultured with tumor cells, the proliferation of TCR0-T cells **(C)** and TCR3-T cells **(D)** in the absence or presence of KHAA. In the absence or presence of KHAA, CellTrace™ Violet pre-stained TCR0- and TCR3-T cells were respectively co-cultured with target cells, including KRAS G12V_8-16_-peptide (5E-6M)-pulsed T2-A1101 cells, KRAS_8-16_-peptide (5E-6M)-pulsed T2-A1101 cells, SW480-A1101 cells or HCC-366 cells, at the E:T ratio of 1:10 or 1:6 for 5 days. The diminished CellTrace™ Violet was tested by flow cytometry and represented the expanded T cells. Error bars indicated the SEM (n=4).

To assess the impact of indoleamine 2,3-dioxygenase (IDO)-derived metabolites on T cell proliferation, we treated TCR0- and TCR3-T cells with mixture (KHAA) of kynurenine and 3-hydroxyanthranilic acid (3-HAA) during co-culture with target cells. KHAA minimally affected TCR3-T cell proliferation. Coculturing with antigen-pulsed T2-A1101 cells (high peptide load (22)), TCR0-T cell expansion was strongly inhibited by KHAA (**Figure 5A**, **Supplementary Figure 11A**); TCR3-T cells maintained robust proliferation, with no significant difference even at 400 μM KHAA (**Figure 5B**, **Supplementary Figure 11B**). Co-culturing with target cells at physiological antigen levels (SW480-A1101 cells), TCR0-T cells failed to proliferate (**Figure 5C**, **Supplementary Figure 12A**); TCR3-T cells showed high expansion, with significant suppression only at ≥200 μM KHAA, but the max inhibition rate was only 15.40% (**Figure 5D**, **Supplementary Figure 12B**). Notably, 12.5 μM KHAA suffices to inhibit CD19-CAR T cells (23). These results demonstrated superior tolerance compared to CAR-T cells. We concluded that TCR3-T cells showed striking resilience to IDO-mediated immunosuppression, outperforming both TCR0-T cells and the CAR-T cells. This was a critical advantage for adoptive cell therapies in IDO-rich TMEs.

### 3.6 Enhanced TGF−β1 resistance in avidity−optimized TCR−T cells sustains antitumor function

**Figure 6:**
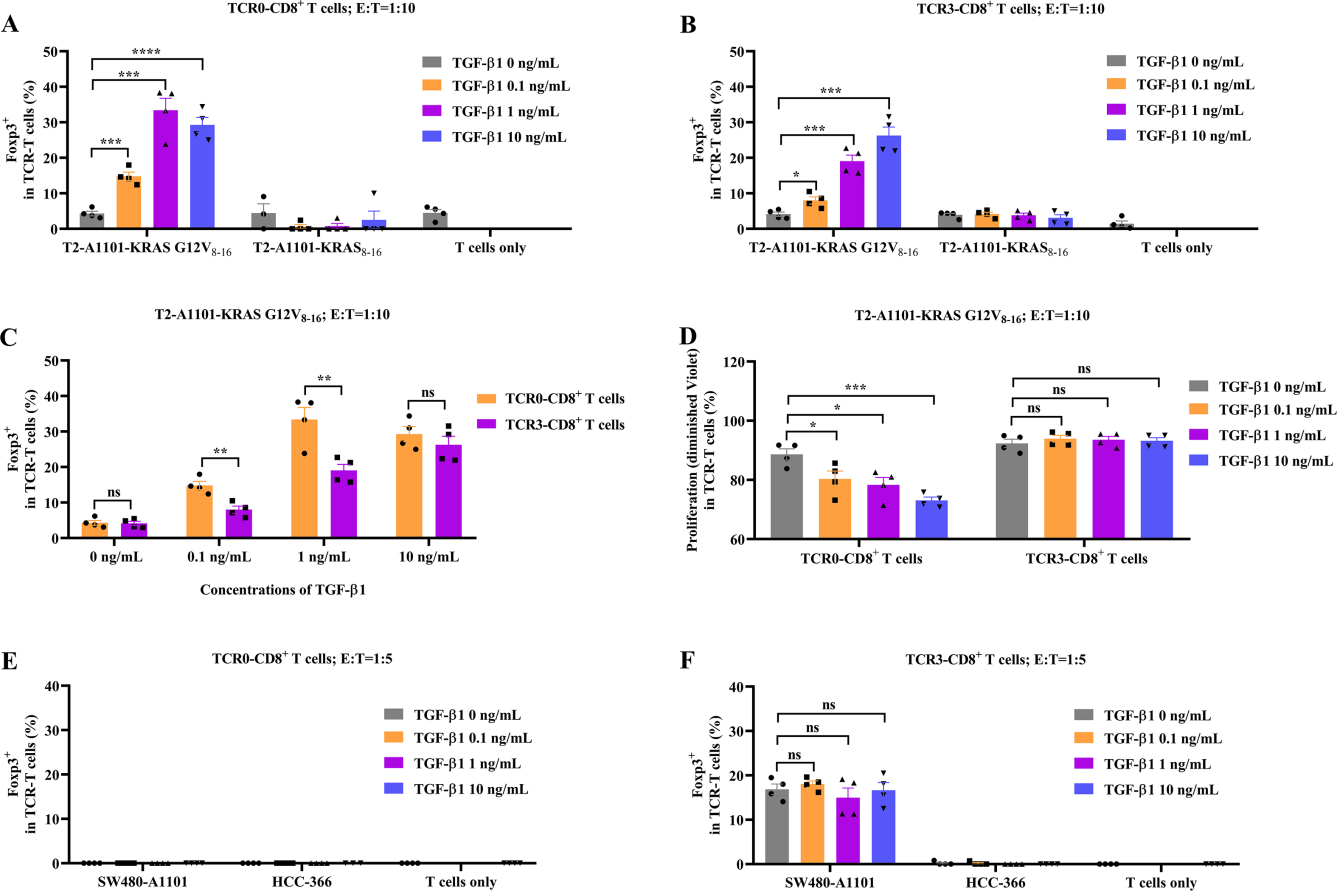
Effect of TGF-β1 on wild-type and optimized TCRs. In the absence or presence of TGF-β1, Foxp3 expression of TCR0-T cells **(A)** and TCR3-T cells **(B)** after recognizing peptide (5E-6M)-pulsed T2-A1101 cells. **(C)** The comparison of the rising trends of Foxp3 expression between TCR0- and TCR3-T cells. **(D)** In the absence or presence of TGF-β1, the proliferation of TCR0- and TCR3-T cells after recognizing KRAS G12V_8-16_-peptide (5E-6M)-pulsed T2-A1101 cells. In the absence or presence of TGF-β1, Foxp3 expression of TCR0-T cells **(E)** and TCR3-T cells **(F)** after recognizing tumor cells. TCR0- and TCR3-T cells were respectively co-cultured with target cells with or without TGF-β1 at the E:T ratio of 1:5 or 1:10 for 5 days. The working concentrations of TGF-β1 were 0.1, 1, and 10 ng/mL. Foxp3 expression and diminished CellTrace™ Violet in T cells were tested by flow cytometry. Error bars indicated the SEM (n=4).

TGF−β1 represents a major barrier to T−cell therapies by promoting regulatory T−cell (Treg) differentiation and suppressing proliferation (24, 25). In this study, we demonstrate that TCR−T cells engineered for higher antigen avidity (TCR3) resisted TGF−β1−mediated immunosuppression and maintained effector function under tumor−like conditions. To assess TGF−β1 sensitivity, TCR0− and TCR3−T cells were co−cultured with target cells across increasing TGF−β1 concentrations. In the absence of neoantigen, both TCR types exhibited minimal baseline Foxp3 expression, which was unaffected by TGF−β1. However, upon stimulation with KRAS G12V_8-16_−pulsed T2−A1101 cells, responses diverged: TCR0−CD8^+^ T cells displayed strong, dose−dependent Foxp3 induction, whereas TCR3−CD8^+^ T cells showed markedly reduced upregulation (**Figures 6A–C**; **Supplementary Figure 13**). Notably, when responding to SW480−A1101 cells, TCR3−CD8^+^ T cells maintained intermediate Foxp3 levels despite TGF−β1 exposure, while TCR0 cells remained Foxp3−negative (**Figures 6E, F**; **Supplementary Figure 14**). This antigen−specific modulation suggested that TCR3 resistance is activation−dependent. Functional assays supported these observations: TCR3−CD8^+^ T cells preserved proliferative capacity under TGF−β1, whereas TCR0-T cells were significantly inhibited (**Figure 6D**, **Supplementary Figure 15**). Together, these results identify TCR-T cell avidity optimization as a strategy to overcome TGF−β1−mediated dysfunction. The dual resistance of TCR3-T cells to both Treg conversion and proliferation arrest underscores its potential for sustained efficacy in immunosuppressive tumor microenvironments.

### 3.7 TCR3-T cells exhibited enhanced tumor infiltration and immune cell recruitment

**Figure 7:**
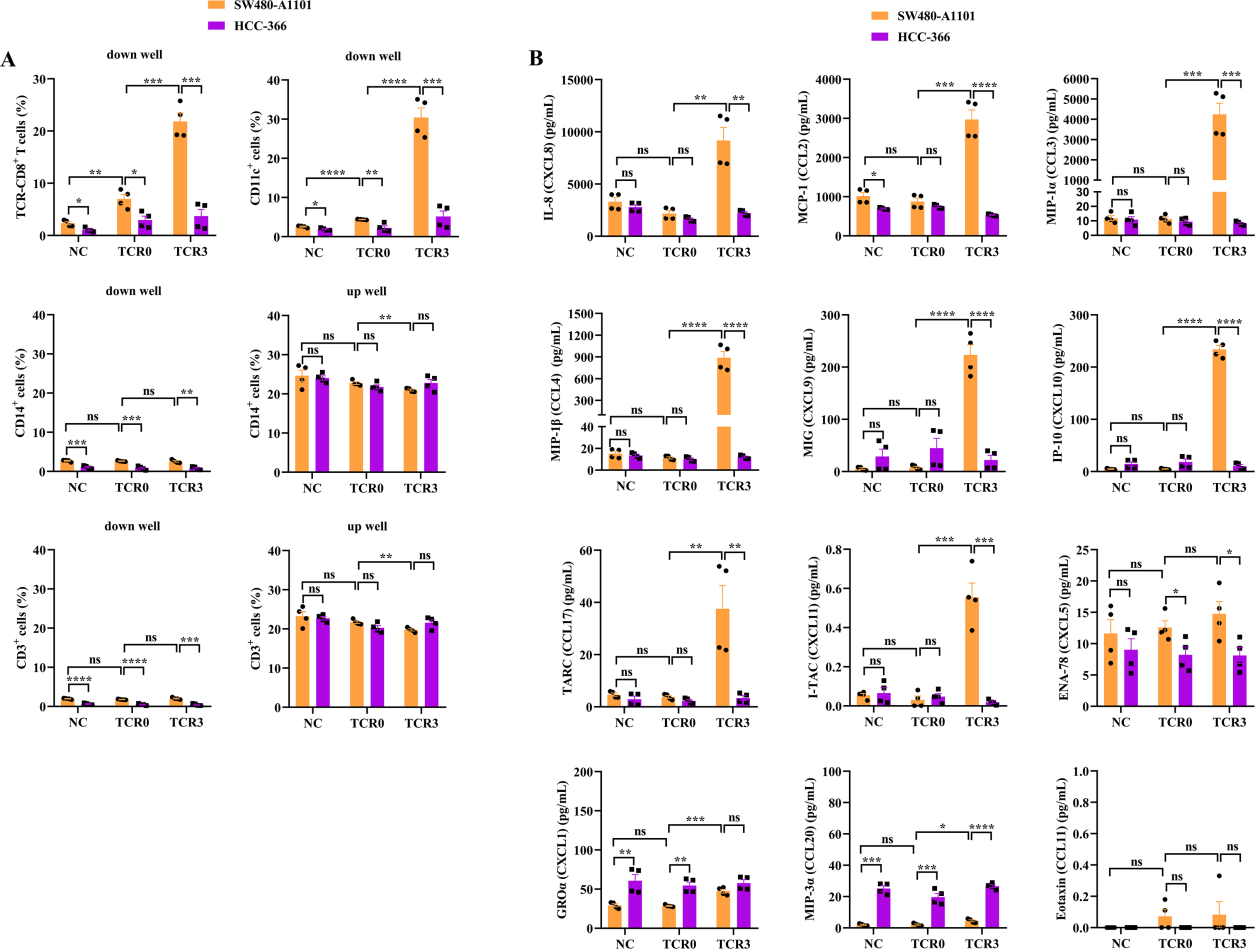
Comparison of infiltration, immune-cell recruitment, and chemokine secretion between wild-type and optimized TCRs. **(A)** The infiltration and immune-cell recruitment. **(B)** The secretion of 12 chemokines. The immune cells were cultured with target cells at the E:T ratio of 1:1 for 3 days in a Transwell plate. The infiltrated TCR-T cells and recruited immune cells in down and up wells were measured by flow cytometry, and the chemokines in down well were tested by LEGENDplex™. Error bars indicated the SEM (n=4).

To assess the migratory capacity and immune-modulatory effects of TCR3-T cells, we employed a transwell co-culture system with TCR-T cells and PBMCs in the upper chamber and target cells below. TCR3-T cells demonstrated superior infiltration and recruitment of CD11c^+^ dendritic cells/macrophages toward antigen-positive SW480-A1101 cells compared to antigen-negative HCC-366 cells or TCR0-T cells (*P* < 0.001; **Figure 7A**; **Supplementary Figure 16**). While TCR0-T cells showed modest infiltration and recruitment relative to SW480-A1101 cells, both metrics significantly exceeded background levels observed with HCC-366 cells or untransduced CD8^+^ T cells (NC) (**Figure 7A**, **Supplementary Figure 16**). Monocyte (CD14^+^) and polyclonal CD3^+^ T cell recruitment was minimal across all groups, though SW480-A1101 cells elicited slightly higher recruitment with TCR3-T and TCR0-T cells compared to HCC-366 cells (**Figure 7A**, **Supplementary Figure 16**). No intergroup differences were observed in the upper chamber, where ~20% of monocytes and CD3^+^ T cells remained (**Figure 7A**, **Supplementary Figure 16**). These results indicated that TCR3-T cells selectively enhanced infiltration and CD11c^+^ cell recruitment upon antigen engagement.

Chemokine profiling revealed mechanistic insights. TCR3-T cells co-cultured with SW480-A1101 cells secreted significantly elevated levels of CXCL8, CCL2, CCL3, and CCL4, along with the T-cell chemoattractants CXCL9, CXCL10, and CCL17 (*P* < 0.01 vs. TCR0-T or HCC-366 controls; **Figure 7B**; **Supplementary Figure 17**). In contrast, CXCL11, CXCL5, CXCL1, CCL20, and CCL11 secretion was negligible and comparable to controls. TCR0-T cells and untransduced CD8^+^ T cells (NC) showed no chemokine induction. This chemokine signature aligned with the observed infiltration and recruitment phenotypes, suggesting TCR3-T cells orchestrate an antigen-dependent immune microenvironment.

### 3.8 TCR3-T cells showed strict antigen specificity without off-target recognition

**Figure 8:**
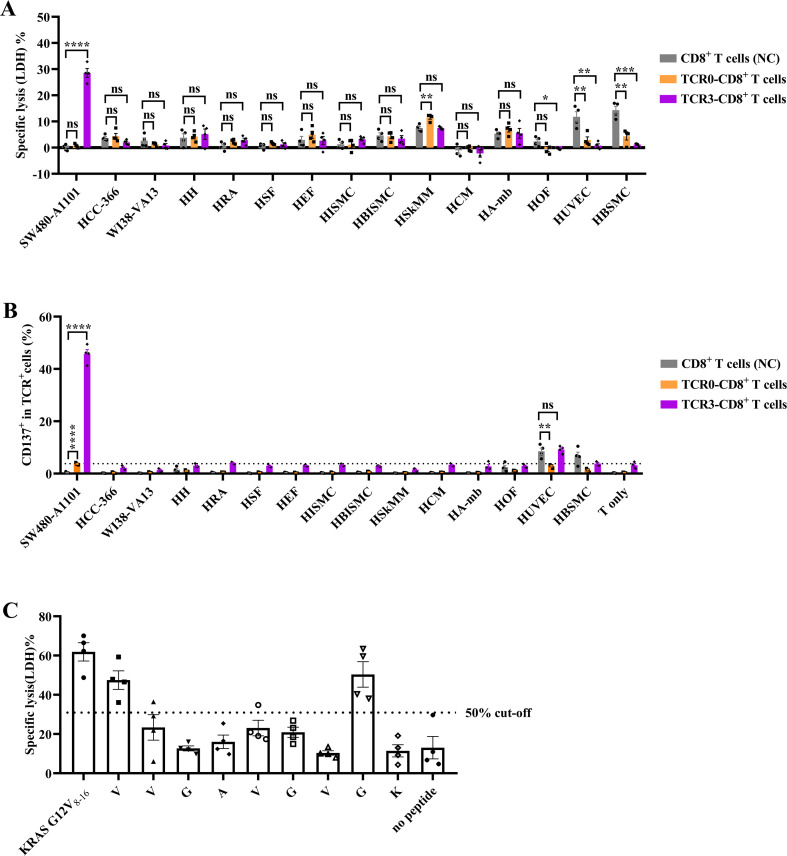
Analysis of co-culture of TCR3-T cells and human primary cells. **(A)** Cytotoxicity of TCR3-T cells on target cells. **(B)** Activation of TCR3-T cells responding to target cells. TCR3-T cells were co-cultured with SW480-A1101 cells, HCC-366 cells and 13 different human primary cells at the E:T ratio of 5:1 for 16 h. The cytotoxicity was determined by the amount of LDH releasing, and activation of T cells was determined by CD137 levels. TCR0-T cells and CD8^+^ T cells were used as control effector cells. **(C)** Alanine scanning assay through cytotoxicity. TCR3-T cells were co-cultured with various peptides (1E-10M)-loaded T2-A1101 cells at the E:T ratio of 5:1 for 16 h. The cytotoxicity was determined with the LDH assay. Error bars indicated the SEM (n=4).

To evaluate off-target reactivity, TCR3-CD8^+^ T cells were co-cultured with 13 primary human cell types, including HUVEC, WI38-VA13, HH, HRA, HSF, HEF, HISMC, HBISMC, HOF, HSkMM, HAmb, HBSMC, and HCM. LDH release assays confirmed potent killing of SW480-A1101 cells but no significant increased cytotoxicity against any primary cell type (*P* > 0.05 vs. untransduced CD8^+^ T cells) (**Figure 8A**). TCR0-T cells showed no activity against SW480-A1101 or primary cells. Activation marker CD137 further validated specificity. TCR3-T cells exhibited robust upregulation of CD137 upon SW480-A1101 recognition but not with HUVECs or other primary cells (background levels; **Figure 8B**, **Supplementary Figure 18**). TCR0-T cells showed minimal CD137 expression. Structural basis of specificity was further detected. Alanine scanning identified seven critical residues (G3, A4, V5, G6, V7, and ancho residues V2, K9) in the KRAS G12V_8–16_ peptide, where substitutions reduced TCR3-T cell cytotoxicity by >50% (**Figure 8C**). Two additional residues (V1, G8) contributed moderately (>15% reduction, **Figure 8C**). This stringent dependence on the KRAS peptide-HLA interface (26) explained the absence of off-target recognition.

## 4 Discussion

The KRAS is an oncogenic driver gene with high-frequency mutations in human cancers. Mutated KRAS persistently activates mitogen-activated protein kinase and phosphatidylinositol 3-kinase pathways, contributing to the formation and maintenance of tumors. Most KRAS mutations occur in codons 12, 13, or 61, resulting from substitutions of single deoxynucleotides (8, 11). Mutated KRAS is an attractive target for cancer therapy. Until two small molecule inhibitors are approved, KRAS mutations are considered undruggable (10). However, immune killing is the preferred method of eliminating cancer. A series of mutated KRAS epitopes, in the nonamer or/and decamer-peptide manner, have been identified to be presented by HLA-I molecules on the tumor cell surface, including G12D in the context of HLA-C*08:02, G12C/D/V in the context of HLA-C*03:04, G12V in the context of HLA-C*03:03 and HLA-C*01:02, G12D/R in the context of HLA-B*07:02, G12C/D/R/V in the context of HLA-A*68:01, HLA-A*11:01 and HLA-A*03:01, G12R/V in the context of HLA-A*30:01, Q61H/L/R in the context of HLA-A*01:01, and G13D in the context of HLA-B*03:01 (11, 27). T cells recognizing most of these epitopes have been isolated from humans or transgenic mice, and exhibit the ability to activate and kill tumor cells *in vitro*; however, the anti-tumor function *in vivo* is not optimal (6, 8, 9, 20, 26, 27). Our discovery of TCR0, a novel human TCR specific for KRAS G12V_8-16_-HLA-A*11:01, provides a lead for addressing this unmet need. HLA-A*11:01 is present in 15-20% of East Asian and 5-10% of Caucasian populations (28), covering a clinically meaningful subset of patients. While TCR0 demonstrated precise KRAS G12V_8–16_ specificity (**Figure 1**), its low intrinsic affinity (KD = 217 μM; **Supplementary Table 6**; **Supplementary Figure 19**) restricted cytotoxicity to target cells pulsed with non-physiological peptide levels (>1 μM) (**Figures 1**, **2**)—a critical limitation given tumor cells typically present ≤1 nM of KRAS peptide-HLA complexes (22, 29). This mirrors the weak binding typical of natural TCR-pHLA interactions (30), explaining why endogenous T cells often fail to control KRAS-mutant tumors. These features limit the potential use of TCR0 as a candidate molecule for clinical drug development.

T-cell immunity is initiated by the interaction between the TCR and peptide-HLA (pHLA). Each TCR variable region contains three CDRs. CDR1 interacts with the termini of the peptide and/or HLA helices, CDR2 loops over the HLA groove-flanking helices, and CDR3 binds to the center of peptides (31). Compared to antibodies without somatic hypermutation, the affinity (K_D_) of natural TCR interacting with pHLA can be low at 500 μM (30). Using yeast or phage display to perform molecular evolution within CDRs, TCR affinity can be enhanced to the nanomolar or picomolar range (31–33). The *in vitro* affinity-enhanced TCR confers a stronger function of tumor killing, but carries the risk of off-target effects (34). However, the approval afamitresgene autoleucel (afami-cel), a TCR-transduced T-cell product with engineered high-affinity TCR against MAGE-A4, and Tebentafusp, an engineered high-affinity TCR/CD3 engager against gp100, both show very good clinical safety profiles (1, 2). In this study, we employed phage display to rationally optimize TCR0’s CDR1α region, which engages both peptide and HLA (31). The resulting TCR3 variant exhibited over 20,000-fold improved functional avidity while maintaining specificity, enabling lysis of HLA-A*11:01^+^ intestinal cancer (SW480-A1101), lung cancer (SHP-77-A1101), and pancreatic cancer (CFPAC-1-A1101) at physiological antigen levels (**Figures 2**, **3**). Alanine scanning confirmed absolute dependence on five peptide residues G3, A4, V5, G6, V7, and two residues V2, K9 anchoring the peptide to HLA-A*11:01 (**Figure 8**), which was consistent with a report by Dan Lu (26). Notably, TCR3 showed no cross-reactivity across 13 primary cell types (**Figure 8**), a critical safety consideration given KRAS’s role in normal tissue homeostasis (35). The high background from four normal cells, including HSkMM, HAmb, HUVEC and HBSMC, may result from the activated allogenic T cells and the higher sensitivity of normal cells (**Figure 8**). Thus, the engineered TCR3 conferred higher avidity and cytotoxicity to T cells and strictly retained its specificity.

In the clinic, owing to interference from the TME, only a small number of patients show a persistent response to immunotherapy (36). TME is an ecosystem that forms during the battle between the tumor and the patient’s body and supports the development, progression, and metastasis of tumors (37). Cancer cells, T cells, B cells, DC, Tregs, myeloid-derived suppressor cells (MDSCs), tumor-associated macrophages (TAMs), cancer-associated fibroblasts (CAFs), tumor vasculature, lymphatics, and adipocytes et al. constitute the cellular components of the TME. Surrounding those cellular components is the extracellular matrix (ECM), which is composed by collagens, adhesive glycoproteins, proteoglycans and integrins, and soluble factors, such as chemokines, cytokines, TGF-β, and IDO (36–38). Infiltrating effector T-cells in the interior of the TME are suppressed by various mechanisms derived from the TME.

PD-L1, an immune “checkpoint” inhibitor, is highly expressed by cancer cells, B cells, DCs, MDSCs, TAMs, and CAFs within the TME, inhibits anti-tumor T cell responses, and induces Tregs dysfunction by interacting with PD-1 (36). Blockade of the PD-L1: PD-1 axis can restore the effector functions of T cells, but simultaneously trigger potent suppressor functions of Tregs, which explains the lack of response to PD-1 blockade immunotherapy in some patients (36). We used a human PD-L1 highly expressed cell line, SW480-A1101-hPD-L1, to analyze the effect of the PD-L1: PD-1 pathway on TCR3-T cells. In the presence of high hPD-L1 levels, TCR3 could still direct CD8^+^ T cells to activate and kill SW480-A1101-hPD-L1 cells *in vitro* and *in vivo*, which was not significantly different from that of wild-type SW480-A1101 cells (**Figure 4**). However, high hPD-L1 levels significantly inhibited the activation and cytotoxicity of TCR0-CD8^+^T cells (**Figure 4**). Unexpectedly, the detection of hPD-1 on TCR3-T cells was significantly lower when co-cultured with high-level hPD-L1 target cells, SW480-A1101-hPD-L1, even though we used a two-fold recommended amount of antibody for detection (**Figure 4**). Thus, the clinical application of TCR3 may not be affected by PD-L1, and without the combination of PD-L1 blockade would not activate the Tregs inhibition in the TME. Furthermore, evaluating the different impact of anti-PD-1 or anti-PD-L1 antibodies blockade on TCR0 and TCR3 is a logical and important next step.

IDO, which is produced by cancer cells and some immune cells, converts tryptophan into kynurenine and 3-HAA, resulting in the suppression of T-cell physiological functions and even the induction of T-cell death (23). We proved that relative to CAR-T cells (23) and TCR0-CD8^+^ T cells, TCR3 granted T cells better tolerance to the immunosuppressive metabolites present in the TME (**Figure 5**).

Within the TME, the TGF-β is abundantly secreted by tumor cells, TAMs and MDSCs. TGF-β downregulates anti-tumor immunity through several mechanisms, including the transformation of T cells into FoxP3^+^ Treg cells and inhibition of T-cell functions. FoxP3^+^ Tregs directly suppress CD8^+^ T cell cytotoxicity (24, 25). *In vitro*, under non-physiological conditions, the higher-avidity feature gave TCR3-CD8^+^ T cells more resistance to TGF-β (**Figure 6**). Under physiological conditions of being co-cultured with tumor cells, TCR3-CD8^+^ T cells expressed low Foxp3 level, but TGF-β1 did not further increase FoxP3 expression in TCR3-CD8^+^ T cells (**Figure 6**). Therefore, in the case of cytotoxic functions, TCR3 may leave T cells unaffected by TGF-β in the TME, and this feature is not present in all TCRs (**Supplementary Figures 2C**, **20**). Interestingly, cytotoxic T cells also weakly expressed FoxP3 in the absence of TGF-β (**Figures 2**, **6**), likely mediated by other factors in tumor cells. In the presence of a small number of FoxP3^+^ cells, TCR3 still granted T cells to show cell-specific killing and proliferation functions against target cells (**Figures 2**, **3**, **5**).

Chemokines belong to a group of chemoattractant cytokines and contain 44 members, that are classified into CC and CXC subfamilies based on the N-terminal domain (39, 40). Under stress conditions, such as radiation, hypoxia, and anti-cancer drugs, cancer cells would produce chemokines to recruit immune cells and promote angiogenesis, tumor growth, and metastasis (41, 42). In this study, we analyzed 12 chemokines from the system of TCR-CD8^+^ T cells attacking target cells, including IL-8 (CXCL8), MCP-1 (CCL2), MIP-1α (CCL3), MIP-1β (CCL4), MIG (CXCL9), IP-10 (CXCL10), TARC (CCL17), I-TAC (CXCL11), ENA-78 (CXCL5), GROα (CXCL1), MIP-3α (CCL20), and Eotaxin (CCL11). The secretion of only six chemokines largely and significantly increased in the system of TCR3-CD8^+^ T cells attacking SW840-A1101 cells (**Figure 7**), including CXCL8 that simultaneously shows the pro-cancer and anti-cancer properties (41), CCL2 and CCL3 that both promote tumor invasion and metastasis and recruit macrophage and neutrophil (39, 41), CCL4 that leads to angiogenesis and recruits macrophages and neutrophils (39), and CXCL9 and CXCL10 that both recruit effector T cells to control tumor growth (36). Meanwhile, owing to the secretion of CXCL9 and CXCL10, TCR3-CD8^+^ T cells significantly infiltrated the tumor site containing the KRAS G12V_8–16_ target (**Figure 7**). Except for tumor-draining lymph nodes, DCs can process and present tumor antigens to T cells within the TME (36). Based on the massive secretion of CCL2, CCL3, and CCL4, CD11c positive cells, which represent conventional DCs and monocyte-derived macrophages (43), were significantly recruited to the site of effector T cell aggregation (**Figure 7**), to remodel the TME.

## 5 Conclusions

Our work provides a blueprint for targeting solid tumors with oncogenic driver mutations: (i) epitope selection based on allele frequency (KRAS G12V) and HLA prevalence (HLA-A*11:01), (ii) structure-guided affinity optimization preserving specificity, and (iii) systematic TME resistance profiling. TCR3, which resulted from the above blueprint, is a promising candidate for clinical immunotherapy against mutated KRAS and will solve the problems regarding clinical effectiveness, safety, and cancer immune escape. Future studies should evaluate TCR3 in primary patient-derived organoids and immunocompetent KRAS G12V models to assess its potential to overcome the stromal barriers characteristic of these malignancies.

## Data Availability

The data supporting the conclusions of this study will be available from the corresponding author upon reasonable request.

## References

[1] Nathan P Hassel JC Rutkowski P Baurain JF Butler MO Schlaak M . Overall survival benefit with tebentafusp in metastatic uveal melanoma. N Engl J Med. (2021) 385:1196–206. doi: 10.1056/NEJMoa2103485, PMID: 34551229

[2] Hong DS Van Tine BA Biswas S McAlpine C Johnson ML Olszanski AJ . Autologous T cell therapy for MAGE-A4(+) solid cancers in HLA-A*02(+) patients: a phase 1 trial. Nat Med. (2023) 29:104–14. doi: 10.1038/s41591-022-02128-z, PMID: 36624315 PMC9873554

[3] de Vries EGE Ruschoff J Lolkema M Tabernero J Gianni L Voest E . Phase II study (KAMELEON) of single-agent T-DM1 in patients with HER2-positive advanced urothelial bladder cancer or pancreatic cancer/cholangiocarcinoma. Cancer Med. (2023) 12:12071–83. doi: 10.1002/cam4.5893, PMID: 37119523 PMC10278525

[4] Lu J Jiang G . The journey of CAR-T therapy in hematological Malignancies. Mol Cancer. (2022) 21:194. doi: 10.1186/s12943-022-01663-0, PMID: 36209106 PMC9547409

[5] Santonocito C Rizza R Paris I Marchis L Paolillo C Tiberi G . Spectrum of germline BRCA1 and BRCA2 variants identified in 2351 ovarian and breast cancer patients referring to a reference cancer hospital of Rome. Cancers (Basel). (2020) 12:02. doi: 10.3390/cancers12051286, PMID: 32438681 PMC7281099

[6] Sim MJW Lu J Spencer M Hopkins F Tran E Rosenberg SA . High-affinity oligoclonal TCRs define effective adoptive T cell therapy targeting mutant KRAS-G12D. Proc Natl Acad Sci U S A. (2020) 117:12826–35. doi: 10.1073/pnas.1921964117, PMID: 32461371 PMC7293613

[7] Douglass J Hsiue EH Mog BJ Hwang MS DiNapoli SR Pearlman AH . Bispecific antibodies targeting mutant RAS neoantigens. Sci Immunol. (2021) 6:02. doi: 10.1126/sciimmunol.abd5515, PMID: 33649101 PMC8141259

[8] Wang QJ Yu Z Griffith K Hanada K Restifo NP Yang JC . Identification of T-cell receptors targeting KRAS-mutated human tumors. Cancer Immunol Res. (2016) 4:204–14. doi: 10.1158/2326-6066.CIR-15-0188, PMID: 26701267 PMC4775432

[9] Poole A Karuppiah V Hartt A Haidar JN Moureau S Dobrzycki T . Therapeutic high affinity T cell receptor targeting a KRAS(G12D) cancer neoantigen. Nat Commun. (2022) 13:5333. doi: 10.1038/s41467-022-32811-1, PMID: 36088370 PMC9464187

[10] Nagasaka M Potugari B Nguyen A Sukari A Azmi AS Ou SI . KRAS Inhibitors- yes but what next? Direct targeting of KRAS- vaccines, adoptive T cell therapy and beyond. Cancer Treat Rev. (2021) 101:102309. doi: 10.1016/j.ctrv.2021.102309, PMID: 34715449

[11] Linette GP Bear AS Carreno BM . Facts and hopes in immunotherapy strategies targeting antigens derived from KRAS mutations. Clin Cancer Res. (2024) 30:2017–24. doi: 10.1158/1078-0432.CCR-23-1212, PMID: 38266167 PMC11094419

[12] Wu X Song W Cheng C Liu Z Li X Cui Y . Small molecular inhibitors for KRAS-mutant cancers. Front Immunol. (2023) 14:1223433. doi: 10.3389/fimmu.2023.1223433, PMID: 37662925 PMC10470052

[13] Dhillon S . Adagrasib: first approval. Drugs. (2023) 83:275–85. doi: 10.1007/s40265-023-01839-y, PMID: 36763320

[14] Tran E Robbins PF Lu YC Prickett TD Gartner JJ Jia L . T-cell transfer therapy targeting mutant KRAS in cancer. N Engl J Med. (2016) 375:2255–62. doi: 10.1056/NEJMoa1609279, PMID: 27959684 PMC5178827

[15] Leidner R Sanjuan Silva N Huang H Sprott D Zheng C Shih YP . Neoantigen T-cell receptor gene therapy in pancreatic cancer. N Engl J Med. (2022) 386:2112–9. doi: 10.1056/NEJMoa2119662, PMID: 35648703 PMC9531755

[16] Hu H Cheng R Wang Y Wang X Wu J Kong Y . Oncogenic KRAS signaling drives evasion of innate immune surveillance in lung adenocarcinoma by activating CD47. J Clin Invest. (2023) 133:02. doi: 10.1172/JCI153470, PMID: 36413402 PMC9843062

[17] Liu C Zheng S Wang Z Wang S Wang X Yang L . KRAS-G12D mutation drives immune suppression and the primary resistance of anti-PD-1/PD-L1 immunotherapy in non-small cell lung cancer. Cancer Commun (Lond). (2022) 42:828–47. doi: 10.1002/cac2.12327, PMID: 35811500 PMC9456691

[18] Coelho MA de Carne Trecesson S Rana S Zecchin D Moore C Molina-Arcas M . Oncogenic RAS signaling promotes tumor immunoresistance by stabilizing PD-L1 mRNA. Immunity. (2017) 47:1083–99.e1086. doi: 10.1016/j.immuni.2017.11.016, PMID: 29246442 PMC5746170

[19] Winograd R Byrne KT Evans RA Odorizzi PM Meyer AR Bajor DL . Induction of T-cell immunity overcomes complete resistance to PD-1 and CTLA-4 blockade and improves survival in pancreatic carcinoma. Cancer Immunol Res. (2015) 3:399–411. doi: 10.1158/2326-6066.CIR-14-0215, PMID: 25678581 PMC4390506

[20] Bear AS Blanchard T Cesare J Ford MJ Richman LP Xu C . Biochemical and functional characterization of mutant KRAS epitopes validates this oncoprotein for immunological targeting. Nat Commun. (2021) 12:4365. doi: 10.1038/s41467-021-24562-2, PMID: 34272369 PMC8285372

[21] Bassan D Gozlan YM Sharbi-Yunger A Tzehoval E Greenstein E Bitan L . Avidity optimization of a MAGE-A1-specific TCR with somatic hypermutation. Eur J Immunol. (2021) 51:1505–18. doi: 10.1002/eji.202049007, PMID: 33835499 PMC8252751

[22] Purbhoo MA Sutton DH Brewer JE Mullings RE Hill ME Mahon TM . Quantifying and imaging NY-ESO-1/LAGE-1-derived epitopes on tumor cells using high affinity T cell receptors. J Immunol. (2006) 176:7308–16. doi: 10.4049/jimmunol.176.12.7308, PMID: 16751374

[23] Ninomiya S Narala N Huye L Yagyu S Savoldo B Dotti G . Tumor indoleamine 2,3-dioxygenase (IDO) inhibits CD19-CAR T cells and is downregulated by lymphodepleting drugs. Blood. (2015) 125:3905–16. doi: 10.1182/blood-2015-01-621474, PMID: 25940712 PMC4473118

[24] Chen C Wang Z Ding Y Qin Y . Tumor microenvironment-mediated immune evasion in hepatocellular carcinoma. Front Immunol. (2023) 14:1133308. doi: 10.3389/fimmu.2023.1133308, PMID: 36845131 PMC9950271

[25] Liang Z Chen W Guo Y Ren Y Tian Y Cai W . Soluble monomeric human programmed cell death-ligand 1 inhibits the functions of activated T cells. Front Immunol. (2023) 14:1133883. doi: 10.3389/fimmu.2023.1133883, PMID: 37266424 PMC10229872

[26] Lu D Chen Y Jiang M Wang J Li Y Ma K . KRAS G12V neoantigen specific T cell receptor for adoptive T cell therapy against tumors. Nat Commun. (2023) 14:6389. doi: 10.1038/s41467-023-42010-1, PMID: 37828002 PMC10570350

[27] Choi J Goulding SP Conn BP McGann CD Dietze JL Kohler J . Systematic discovery and validation of T cell targets directed against oncogenic KRAS mutations. Cell Rep Methods. (2021) 1:100084. doi: 10.1016/j.crmeth.2021.100084, PMID: 35474673 PMC9017224

[28] Available online at: http://www.allelefrequencies.net/ (Accessed August 11, 2024).

[29] Bossi G Gerry AB Paston SJ Sutton DH Hassan NJ Jakobsen BK . Examining the presentation of tumor-associated antigens on peptide-pulsed T2 cells. Oncoimmunology. (2013) 2:e26840. doi: 10.4161/onci.26840, PMID: 24482751 PMC3894244

[30] Bridgeman JS Sewell AK Miles JJ Price DA Cole DK . Structural and biophysical determinants of alphabeta T-cell antigen recognition. Immunology. (2012) 135:9–18. doi: 10.1111/j.1365-2567.2011.03515.x, PMID: 22044041 PMC3246648

[31] Dunn SM Rizkallah PJ Baston E Mahon T Cameron B Moysey R . Directed evolution of human T cell receptor CDR2 residues by phage display dramatically enhances affinity for cognate peptide-MHC without increasing apparent cross-reactivity. Protein Sci. (2006) 15:710–21. doi: 10.1110/ps.051936406, PMID: 16600963 PMC2242494

[32] Li Y Moysey R Molloy PE Vuidepot AL Mahon T Baston E . Directed evolution of human T-cell receptors with picomolar affinities by phage display. Nat Biotechnol. (2005) 23:349–54. doi: 10.1038/nbt1070, PMID: 15723046

[33] Holler PD Holman PO Shusta EV O’Herrin S Wittrup KD Kranz DM . *In vitro* evolution of a T cell receptor with high affinity for peptide/MHC. Proc Natl Acad Sci U S A. (2000) 97:5387–92. doi: 10.1073/pnas.080078297, PMID: 10779548 PMC25838

[34] Linette GP Stadtmauer EA Maus MV Rapoport AP Levine BL Emery L . Cardiovascular toxicity and titin cross-reactivity of affinity-enhanced T cells in myeloma and melanoma. Blood. (2013) 122:863–71. doi: 10.1182/blood-2013-03-490565, PMID: 23770775 PMC3743463

[35] Hill W Zaragkoulias A Salvador-Barbero B Parfitt GJ Alatsatianos M Padilha A . EPHA2-dependent outcompetition of KRASG12D mutant cells by wild-type neighbors in the adult pancreas. Curr Biol. (2021) 31:2550–60.e2555. doi: 10.1016/j.cub.2021.03.094, PMID: 33891893 PMC8231095

[36] Yenyuwadee S Aliazis K Wang Q Christofides A Shah R Patsoukis N . Immune cellular components and signaling pathways in the tumor microenvironment. Semin Cancer Biol. (2022) 86:187–201. doi: 10.1016/j.semcancer.2022.08.004, PMID: 35985559 PMC10735089

[37] Baghy K Ladanyi A Reszegi A Kovalszky I . Insights into the tumor microenvironment-components, functions and therapeutics. Int J Mol Sci. (2023) 24:12. doi: 10.3390/ijms242417536, PMID: 38139365 PMC10743805

[38] Osipov A Saung MT Zheng L Murphy AG . Small molecule immunomodulation: the tumor microenvironment and overcoming immune escape. J Immunother Cancer. (2019) 7:224. doi: 10.1186/s40425-019-0667-0, PMID: 31439034 PMC6704558

[39] Korbecki J Kojder K Siminska D Bohatyrewicz R Gutowska I Chlubek D . CC chemokines in a tumor: A review of pro-cancer and anti-cancer properties of the ligands of receptors CCR1, CCR2, CCR3, and CCR4. Int J Mol Sci. (2020) 21:13. doi: 10.3390/ijms21218412, PMID: 33182504 PMC7665155

[40] Korbecki J Kojder K Kapczuk P Kupnicka P Gawronska-Szklarz B Gutowska I . The effect of hypoxia on the expression of CXC chemokines and CXC chemokine receptors-A review of literature. Int J Mol Sci. (2021) 22:13. doi: 10.3390/ijms22020843, PMID: 33467722 PMC7830156

[41] Nagarsheth N Wicha MS Zou W . Chemokines in the cancer microenvironment and their relevance in cancer immunotherapy. Nat Rev Immunol. (2017) 17:559–72. doi: 10.1038/nri.2017.49, PMID: 28555670 PMC5731833

[42] Zhang W Wang H Sun M Deng X Wu X Ma Y . CXCL5/CXCR2 axis in tumor microenvironment as potential diagnostic biomarker and therapeutic target. Cancer Commun (Lond). (2020) 40:69–80. doi: 10.1002/cac2.12010, PMID: 32237072 PMC7163794

[43] Helft J Bottcher J Chakravarty P Zelenay S Huotari J Schraml BU . GM-CSF mouse bone marrow cultures comprise a heterogeneous population of CD11c(+)MHCII(+) macrophages and dendritic cells. Immunity. (2015) 42:1197–211. doi: 10.1016/j.immuni.2015.05.018, PMID: 26084029

