# Supplementary material for: Avidity-optimized TCR-T cells target KRAS neoantigens for potent cancer clearance and tumor microenvironment remodeling
Source: Front Immunol. 2026 Jan 26;17:1736294. doi: 10.3389/fimmu.2026.1736294 (PMC12883752; doi:10.3389/fimmu.2026.1736294)
Supplement: Supplementary file 1 [file DataSheet1.docx]

Supplementary Material

# Supplementary Figures and Tables

## Supplementary Tables

**Supplementary Table 1.** The details of cell lines

| **Cell Line** | **Brand** | **Catalog** | **Medium** | **Transduction** |
| --- | --- | --- | --- | --- |
| T2 | ATCC | CRL-1992 | Roswell Park Memorial Institute-1640 (Gibco, cat# C11875500BT) | HLA-A*11:01 |
| SW480 | Cobioer, China | CBP60019 | Dulbecco's modiﬁed Eagle's medium (Gibco, cat# C11995500BT) | HLA-A*11:01; hPD-L1 |
| CFPAC-1 | Cobioer, China | CBP60665 | Iscove's Modified Dulbecco's medium (Gibco, cat# C12440500BT) | HLA-A*11:01 |
| SHP-77 | Cobioer, China | CBP60151 | Roswell Park Memorial Institute-1640 (Gibco, cat# C11875500BT) | HLA-A*11:01 |
| HCC-366 | Cobioer, China | CBP60099 | Roswell Park Memorial Institute-1640 (Gibco, cat# C11875500BT) | N/A |
| SW620 | Cobioer, China | CBP60036 | Dulbecco's modiﬁed Eagle's medium (Gibco, cat# C11995500BT) | N/A |

**Supplementary Table 2.** The details of normal cells

| **Normal Cells** | **Brand** | **Catalog** | **Medium** | **Transduction** |
| --- | --- | --- | --- | --- |
| HUVEC | Cobioer, China | CBP60340 | bronchial epithelial cell growth medium (Lonza Bioscience, Switzerland, cat# CC-3170) | N/A |
| WI38-VA13 | Cobioer, China | CBP60974 | Minimum Essential medium (Gibco, cat# 11140050) | HLA-A*11:01 |
| human hepatocytes (HH) | ScienCell | 5200 | hepatocyte medium (ScienCell, cat# 5201) | HLA-A*11:01 |
| human retinal astrocytes (HRA) | ScienCell | 1870 | astrocyte medium (ScienCell, cat# 1801) | HLA-A*11:01 |
| human splenic fibroblasts (HSF) | ScienCell | 5530 | fibroblast medium (ScienCell, cat# 2301) | HLA-A*11:01 |
| human esophageal fibroblasts (HEF) | ScienCell | 2730 | fibroblast medium (ScienCell, cat# 2301) | HLA-A*11:01 |
| human intestinal smooth muscle cells (HISMC) | ScienCell | 2910 | smooth muscle cell medium (ScienCell, cat# 1101) | HLA-A*11:01 |
| human bladder smooth muscle cells (HBISMC) | ScienCell | 4310 | smooth muscle cell medium (ScienCell, cat# 1101) | HLA-A*11:01 |
| human ovarian fibroblasts (HOF) | ScienCell | 7330 | fibroblast medium (ScienCell, cat# 2301) | N/A |
| human skeletal muscle myoblasts (HSkMM) | ScienCell | 3520 | skeletal muscle cell medium (ScienCell, cat# 3501) | HLA-A*11:01 |
| human astrocytes-midbrain (HAmb) | ScienCell | 1850 | astrocyte medium (ScienCell, cat# 1801) | HLA-A*11:01 |
| Human bronchial smooth muscle cells (HBSMC) | PromoCell | C-12561 | smooth muscle cell medium (ScienCell, cat# 1101) | N/A |
| human cardiac myocytes (HCM) | PromoCell | C-12810 | myocyte growth medium (Promocell, cat# C-22070) | HLA-A*11:01 |

**Supplementary Table 3.** The details of primers

| **Primer** | **Sequence** |
| --- | --- |
| YW1 | 5′-atcggtgatgtcggcgatataggcg-3′ |
| YW2 | 5′-gctgtaggtgcagttgatgctaacggt-3′ |
| YW3 | 5′-atcaactgcacctacagcNNKNNKNNKNNKNNKagcttcttttggtaccgtcaatatc-3′ |
| YW4 | 5′-atcaactgcacctacagcgacNNKNNKNNKNNKNNKttcttttggtaccgtcaatatc-3′ |
| YW5 | 5′-tcagcttcctttcgggctttgttagc-3′ |

**Supplementary Table 4.** The details of antibodies

| **Antibody** | **Brand** | **Catalog** |
| --- | --- | --- |
| APC anti-human CD8 Antibody | BioLegend | 344722 |
| PE/Cyanine7 anti-human CD137 (4-1BB) Antibody | BioLegend | 309818 |
| APC anti-mouse TCRβ chain | BioLegend | 109212 |
| APC/Cyanine7 anti-human CD4 Antibody | BioLegend | 300518 |
| CellTrace™ Violet | Invitrogen | C34557 |
| FITC anti-mouse TCR β chain | BioLegend | 109206 |
| Alexa Fluor^®^ 700 anti-human CD14 | BioLegend | 325614 |
| PE/Cyanine7 anti-human CD11c | BioLegend | 337216 |
| APC anti-human PD-L1 | BioLegend | 329708 |
| PE anti-human PD-1 | BioLegend | 379210 |
| Tetramer-PE | *in house* | N/A |
| FITC Rabbit Anti-Active Caspase-3 | BD Pharmingen | 560901 |
| PE anti-human forkhead family transcription factor 3 (Foxp3) Recombinant Antibody | BioLegend | 364704 |
| Alexa Fluor^®^ 647 anti-human forkhead family transcription factor 3 (Foxp3) Recombinant Antibody | BioLegend | 320114 |
| PE anti-human CD8 Antibody | BioLegend | 344706 |
| APC anti-human CD3 Antibody | BioLegend | 317318 |
| PE Streptavidin (SA-PE) | BioLegend | 405204 |

**Supplementary Table 5.** The details of peptides

| **Peptide** | **Sequence** |
| --- | --- |
| parental peptide (KRAS G12V_8-16_) | VVGAVGVGK |
| the first substituted peptide | AVGAVGVGK |
| the second substituted peptide | VAGAVGVGK |
| the third substituted peptide | VVAAVGVGK |
| the fourth substituted peptide | VVGGVGVGK |
| the fifth substituted peptide | VVGAAGVGK |
| the sixth substituted peptide | VVGAVAVGK |
| the seventh substituted peptide | VVGAVGAGK |
| the eighth substituted peptide | VVGAVGVAK |
| the ninth substituted peptide | VVGAVGVGA |

**Supplementary Table 6.** The affinity parameters of TCR0 tested by Surface Plasmon Resonance

| Clone no. | pMHC | ka (1/Ms) | kd (1/s) | K_D_ (M) |
| --- | --- | --- | --- | --- |
| TCR0 | KRAS G12V_8-16_-HLA-A*11:01 | 1.144E+04 | 2.483E+00 | 2.170E-04 |


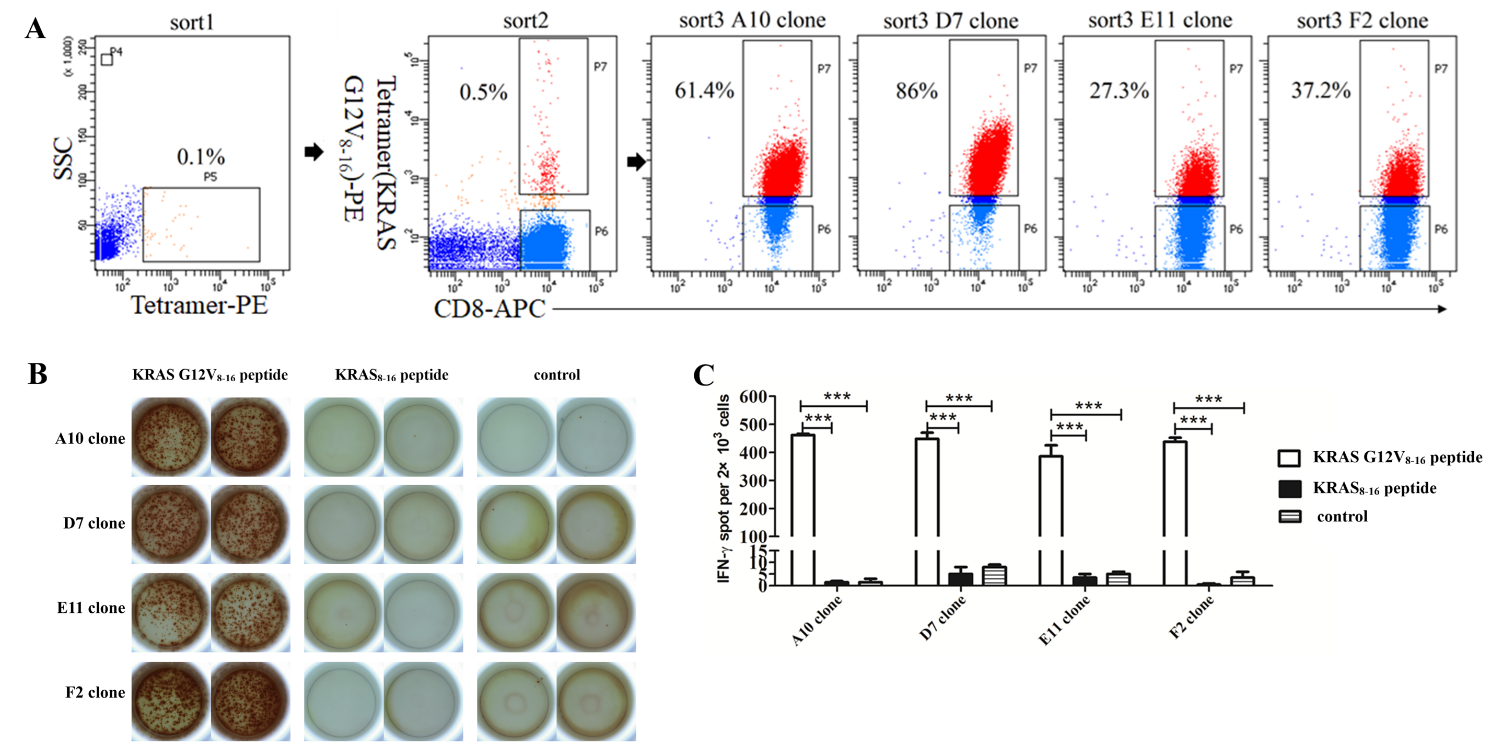


**Supplementary Figure 1. The discovery and functional identification of TCR0**

**(A)** Isolation of KRAS G12V_8-16_-HLA-A*11:01 specific CD8^+^ T-cell clones. After the two rounds of sorting then single-cell expansion, four clones which grew well were selected to analyze the positive rate (KRAS G12V₈‑₁₆–HLA-A*11:01 tetramer) then sorted for functional identification and sequence. Those four clones were respectively A10 clone, D7 clone, E11 clone, and F2 clone. The TCR from A10 clone was designated as TCR0. **(B)** The IFN-γ secretion from four T-cell clones. Four T-cell clones, including A10 clone (TCR0), D7 clone, E11 clone, and F2 clone, were respectively co-cultured with T2 cells at an effector‑to‑target (E:T) ratio of 1:10 (2000:20000) for 16 h in the ELISPOT plate pre-coated with anti-IFN-γ antibody. T2 cells were respectively loaded with KRAS G12V_8-16_ peptide (1E-5M), KRAS_8-16_ peptide (1E-5M) or no peptide (control). After cells being lysed, the biotinylated anti-IFN-γ antibody and SA-HRP were successively incubated. The spots were colored by AEC substrate reagent and counted with AID iSpot Reader Spectrum (AID GmbH). **(C)** The statistical data of IFN-γ secretion from (B). Error bars indicated SEM. Unpaired student's t-test, *P < 0.05; **P < 0.01; ***P < 0.001; comparison was shown by brackets.


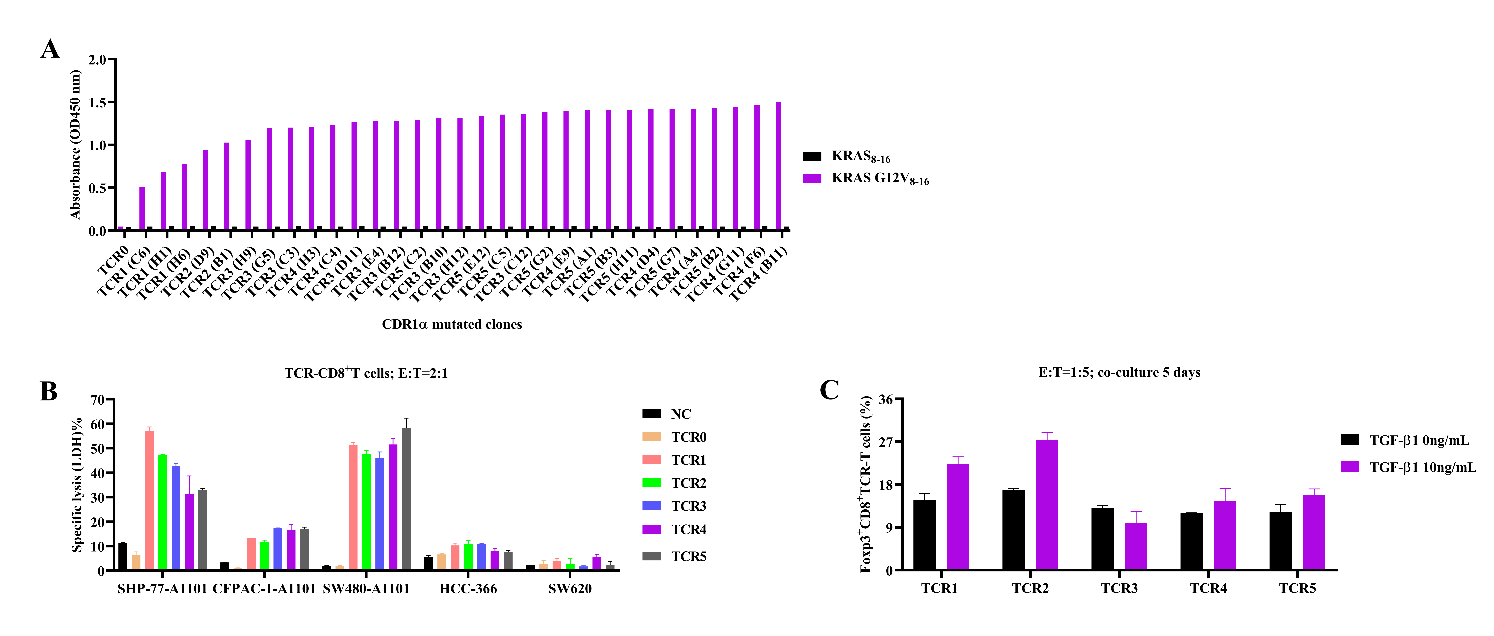


**Supplementary Figure 2. Functional characterization of TCR variants from the CDR1α library**

**(A)** Absorbance values of phage ELISA of 31 TCR variants from the CDR1α library. Following a third round of bio‑panning against the KRAS G12V₈‑₁₆–HLA‑A11:01 complex, 90 colonies from the library were selected and tested for binding to either the KRAS G12V₈‑₁₆–HLA‑A11:01 complex or the wild‑type KRAS₈‑₁₆–HLA‑A*11:01 complex by ELISA. Compared with TCR0, 31 variants showed specifically higher absorbance signals and were subsequently sequenced. This yielded five distinct TCR sequences, designated TCR1 through TCR5. **(B)** Cytotoxicity comparison of five TCR‑CD8^+^ T cell lines against tumor cells. TCR‑CD8^+^ T cells were co‑cultured with target cells at an effector‑to‑target (E:T) ratio of 2:1 for 16 h, and cytotoxicity was assessed by LDH release. **(C)** Comparison of Foxp3 expression in TCR‑CD8^+^ T cells. TCR‑CD8^+^ T cells were co‑cultured with SW480‑A1101 cells at an E:T ratio of 1:5 for 5 days in the presence of TGF‑β1 (0 or 10 ng/mL). Foxp3 expression in TCR‑CD8^+^ T cells was measured by flow cytometry.


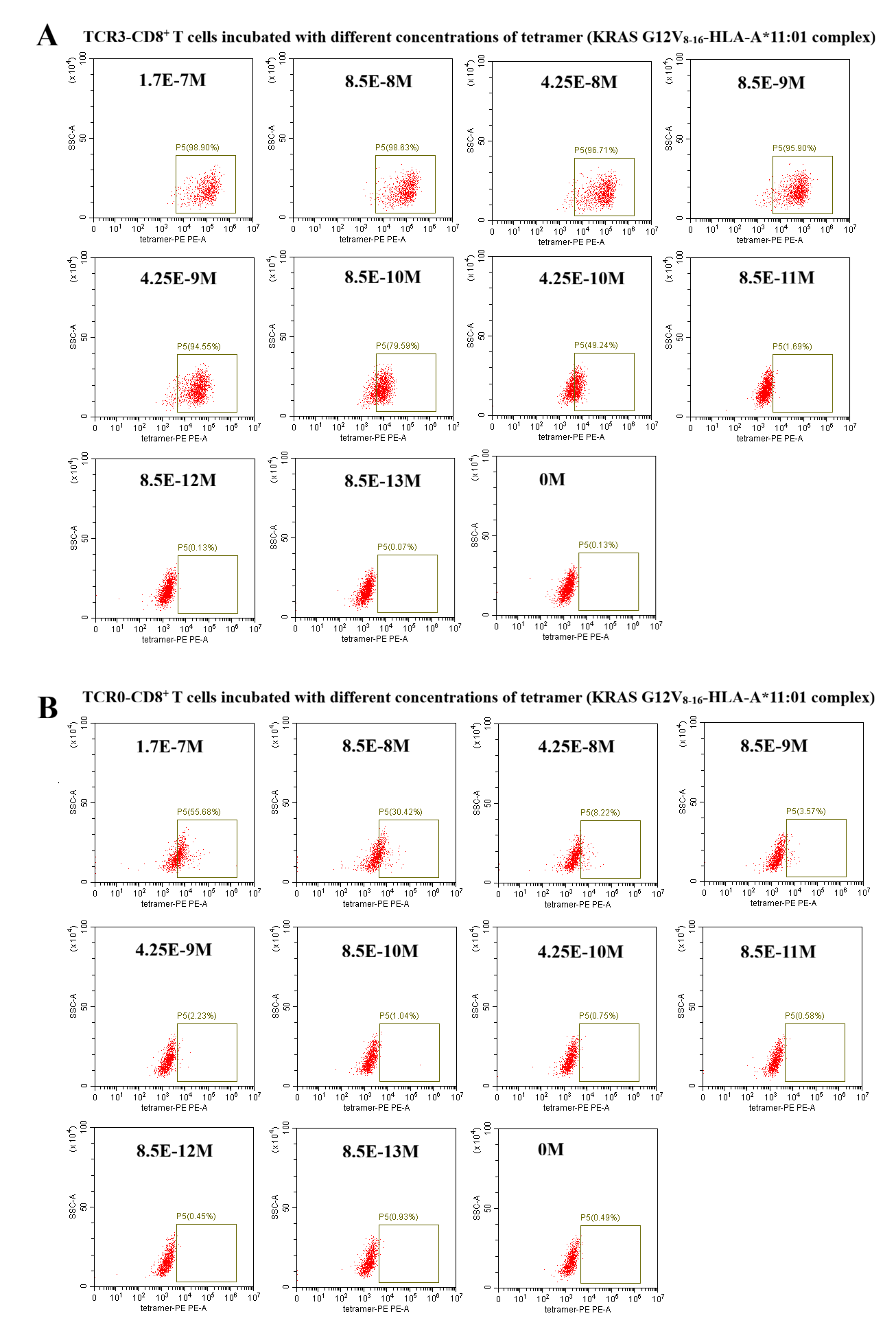


**Supplementary Figure 3. The representative FACS data exhibited the proportion of tetramer positive cells**

**(A)** The proportion of tetramer positive cells from TCR3-CD8^+^ T cells. **(B)** The proportion of tetramer positive cells from TCR0-CD8^+^ T cells. Monomer KRAS G12V_8-16_-HLA-A*11:01 complex was incubated with SA-PE at a ratio of 4:1 to form the tetramer. The concentrations of monomer KRAS G12V_8-16_-HLA-A*11:01 complex tetramer were presented in the graphics. 0.8×10^5^ TCR-T cells in 100 μL PBS (containing 5% FBS) were incubated with the series concentration of tetramer for one hour at 4℃, then added FITC anti-mouse TCR β chain (1 μL) for another 30 min at 4℃. After twice washing with PBS, TCR-T cells were suspended with 100 μL PBS and added DAPI (1 μL). After incubation for 15 min, TCR-T cells were analyzed by ﬂow cytometer (CytoFLEX S, Beckman Coulter).


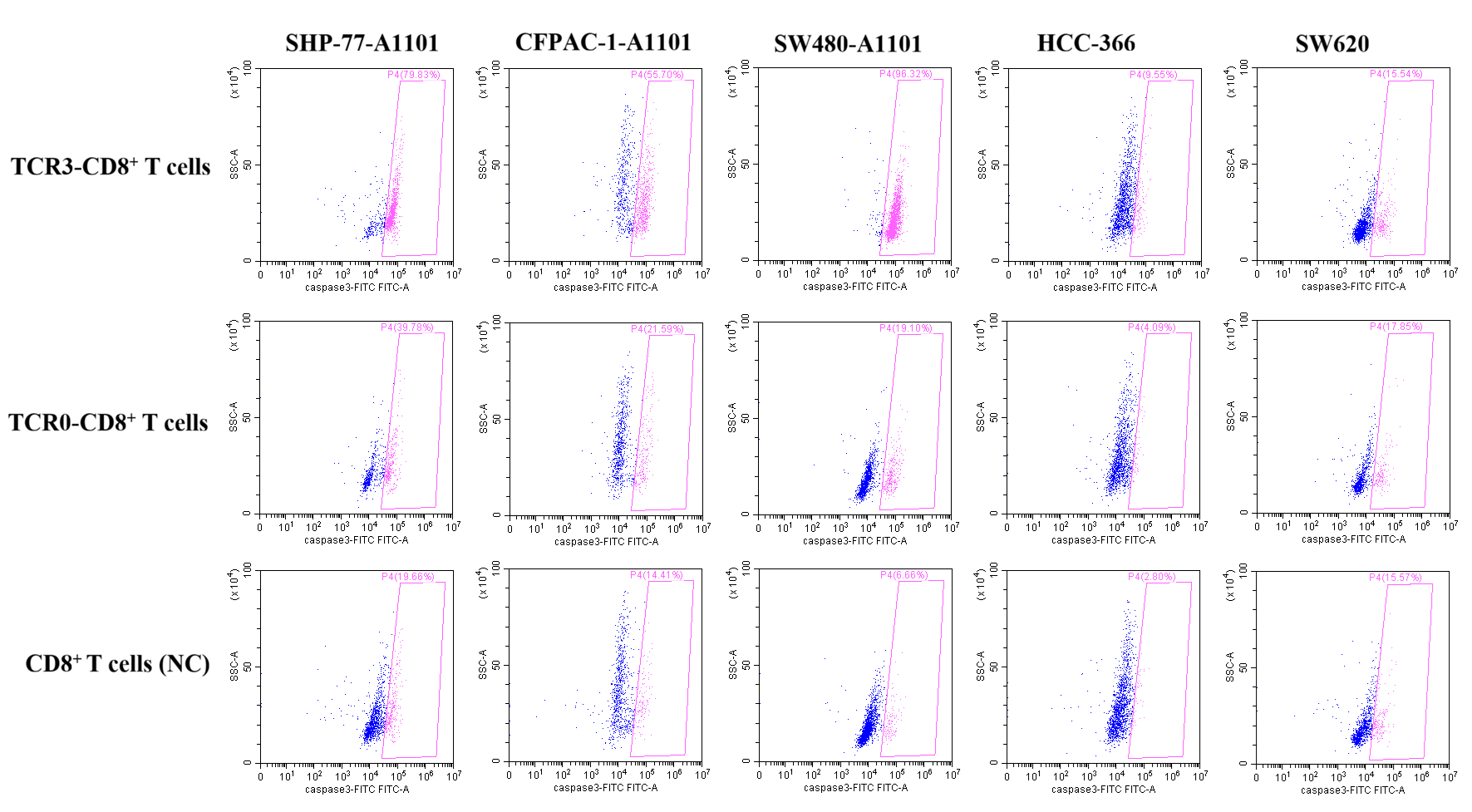


**Supplementary Figure 4. The representative FACS data exhibited the proportion of caspase3 positive tumor cells**

TCR3-CD8^+^ T cells, TCR0-CD8^+^ T cells or CD8^+^ T cells (NC) were respectively co-cultured with SHP-77-A1101 cells, CFPAC-1-A1101 cells, SW480-A1101 cells, HCC-366 cells or SW620 cells at the E:T ratio of 5:1 for 16 h. Cells were firstly surface stained with APC anti-human CD8 Antibody (1:100), then fixed and permeabilized with Fixation/Permeabilization solution (BD biosciences, Cat# 554714), following intracellular stained with FITC Rabbit Anti-Active Caspase-3 (1:20). The active caspase3 rates from tumor cells which were CD8 negative were analyzed by ﬂow cytometer (CytoFLEX S, Beckman Coulter).


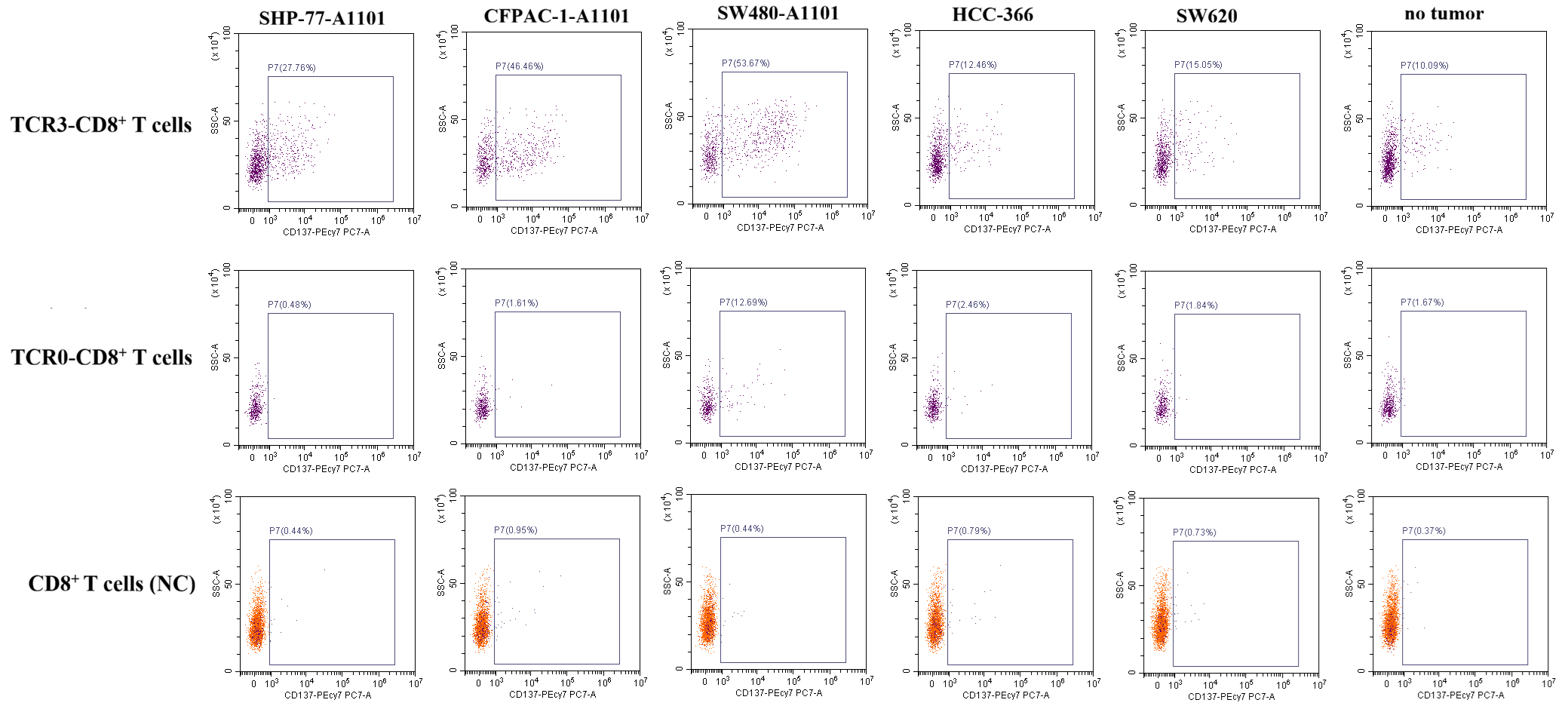


**Supplementary Figure 5. The representative FACS data exhibited the proportion of CD137 positive CD8^+^ T cells**

TCR3-CD8^+^ T cells, TCR0-CD8^+^ T cells or CD8^+^ T cells (NC) were respectively co-cultured with SHP-77-A1101 cells, CFPAC-1-A1101 cells, SW480-A1101 cells, HCC-366 cells, SW620 cells or no tumor at the E:T ratio of 2:1 for 16 h. Cells were simultaneously stained with APC anti-human CD8 Antibody (1:100), FITC anti-mouse TCR β chain (1:100), and PE/Cyanine7 anti-human CD137 (4-1BB) Antibody (1:100). The CD137 rates from TCR-T cells or CD8^+^ T cells, which were DAPI negative, were analyzed by ﬂow cytometer (CytoFLEX S, Beckman Coulter).


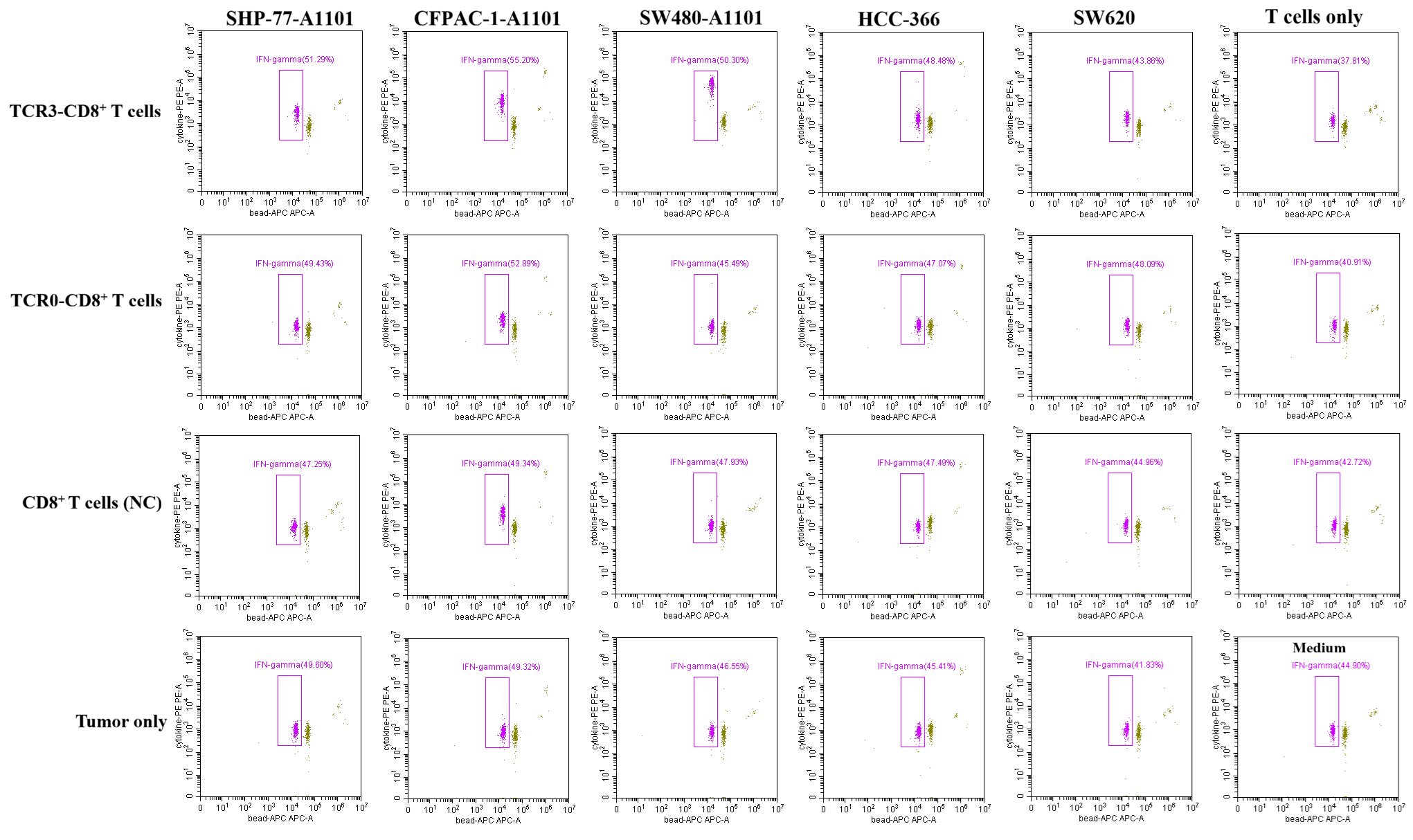


**Supplementary Figure 6. The representative FACS data exhibited the fluorescence intensity of IFN-γ from various groups**

TCR3-CD8^+^ T cells, TCR0-CD8^+^ T cells or CD8^+^ T cells (NC) were respectively co-cultured with SHP-77-A1101 cells, CFPAC-1-A1101 cells, SW480-A1101 cells, HCC-366 cells, or SW620 cells at the E:T ratio of 5:1 for 16 h. “T cells only” group, “Tumor only” group and medium group were used as the control groups and background values. Secreted IFN-γ from supernatant was tested by LEGENDplex™ HU Th1 Panel according to the manufacturer’s instructions. The fluorescence signal of IFN-γ was analyzed through ﬂow cytometer (CytoFLEX S, Beckman Coulter).


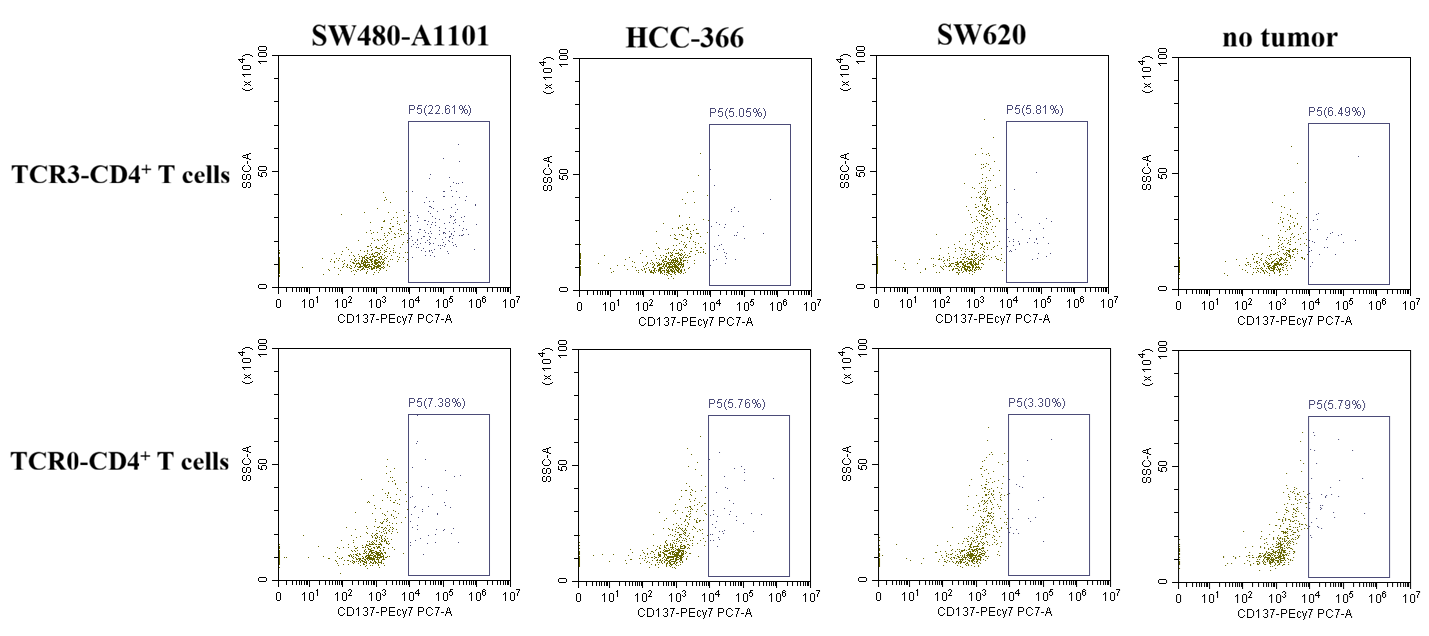


**Supplementary Figure 7. The representative FACS data exhibited the proportion of CD137 positive CD4^+^ TCR-T cells**

TCR3-CD4^+^ T cells or TCR0-CD4^+^ T cells were respectively co-cultured with SW480-A1101 cells, HCC-366 cells, SW620 cells or no tumor at an E:T ratio of 5:1 for 16 h. Cells were simultaneously stained with FITC anti-mouse TCR β chain (1:100) and PE/Cyanine7 anti-human CD137 (4-1BB) Antibody (1:100). The CD137 rates from TCR-T cells, which were DAPI negative, were analyzed by ﬂow cytometer (CytoFLEX S, Beckman Coulter).


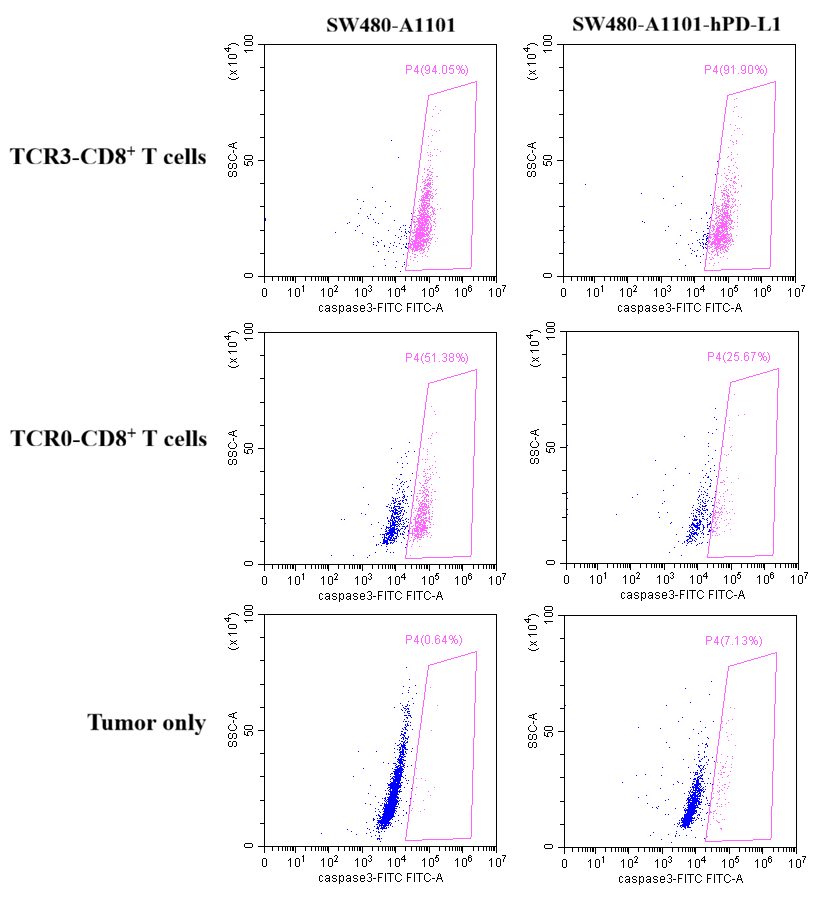


**Supplementary Figure 8. The representative FACS data exhibited the proportion of caspase3 positive tumor cells**

TCR3-CD8^+^ T cells or TCR0-CD8^+^ T cells were respectively co-cultured with SW480-A1101 cells or SW480-A1101-hPD-L1 cells at the E:T ratio of 5:1 for 16 h. Cells were firstly surface stained with APC anti-human CD8 Antibody (1:100), then fixed and permeabilized with Fixation/Permeabilization solution, following intracellular stained with FITC Rabbit Anti-Active Caspase-3 (1:20). The active caspase3 rates from tumor cells, which were CD8 negative, were analyzed by ﬂow cytometer (CytoFLEX S, Beckman Coulter).


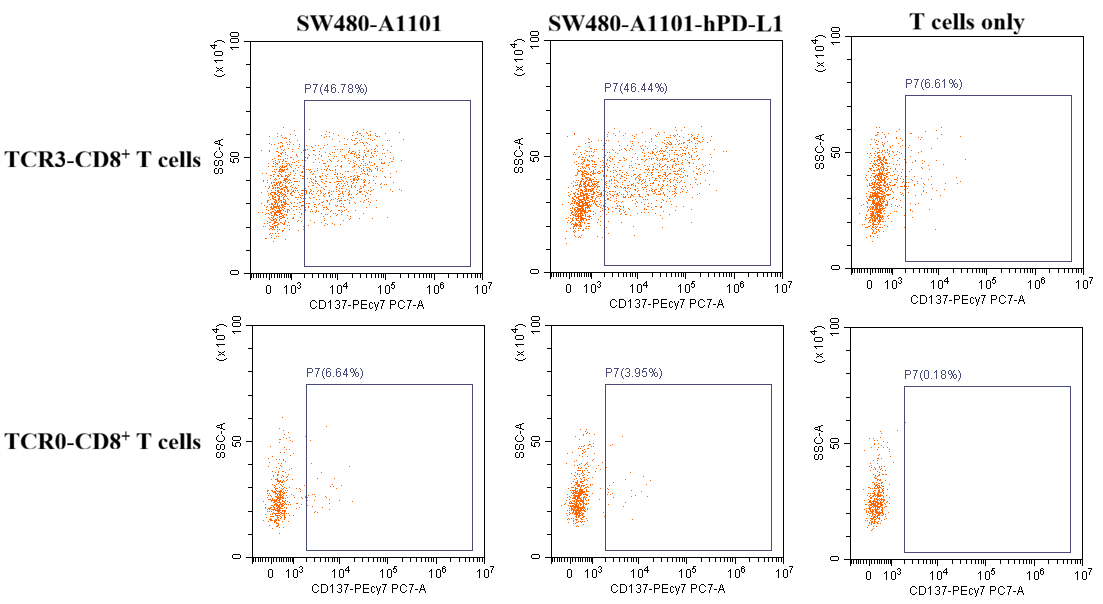


**Supplementary Figure 9. The representative FACS data exhibited the proportion of CD137 positive TCR-T cells**

TCR3-CD8^+^ T cells or TCR0-CD8^+^ T cells were respectively co-cultured with SW480-A1101 cells or SW480-A1101-hPD-L1 cells at an E:T ratio of 2:1 for 16 h. Cells were simultaneously stained with FITC anti-mouse TCR β chain (1:100) and PE/Cyanine7 anti-human CD137 (4-1BB) Antibody (1:100). The CD137 rates from TCR-T cells, which were DAPI negative, were analyzed by ﬂow cytometer (CytoFLEX S, Beckman Coulter).


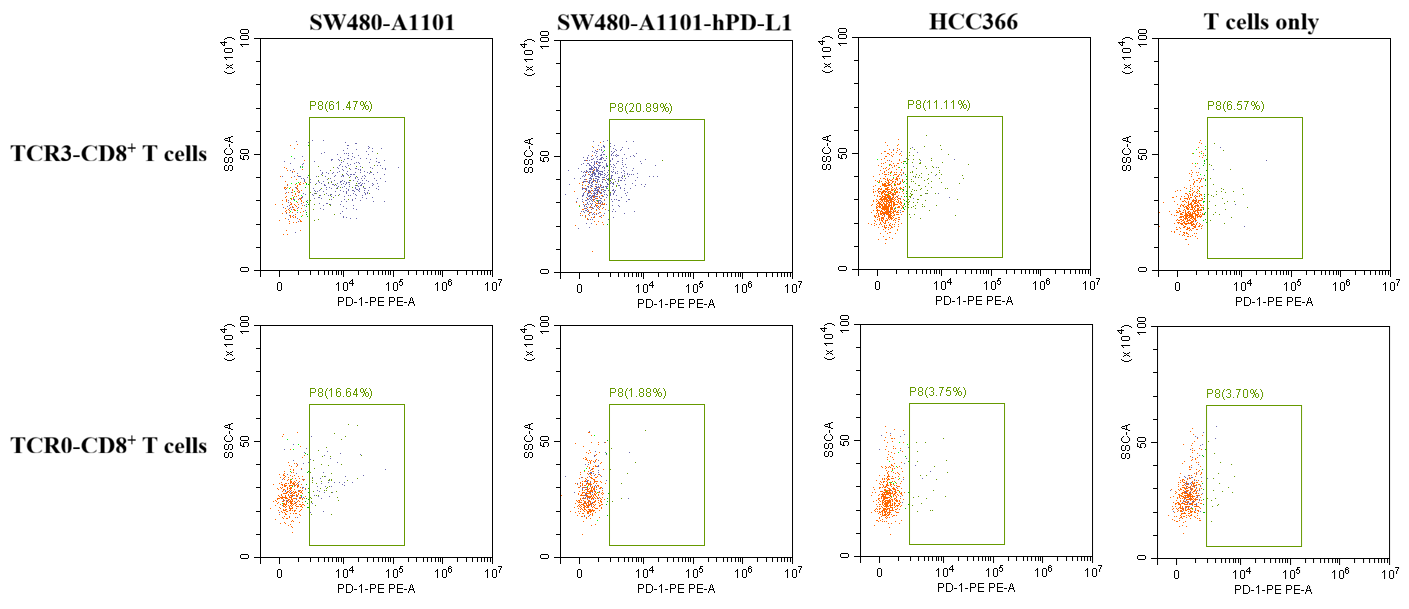


**Supplementary Figure 10. The representative FACS data exhibited the proportion of PD-1 positive CD8^+^ TCR-T cells**

TCR3-CD8^+^ T cells or TCR0-CD8^+^ T cells were respectively co-cultured with SW480-A1101 cells, SW480-A1101-hPD-L1 cells or HCC366 cells at an E:T ratio of 2:1 for 16 h. Cells were simultaneously stained with FITC anti-mouse TCR β chain (1:100) and PE anti-human PD-1 (1:20). The PD-1 rates from TCR-T cells, which were DAPI negative, were analyzed by ﬂow cytometer (CytoFLEX S, Beckman Coulter).


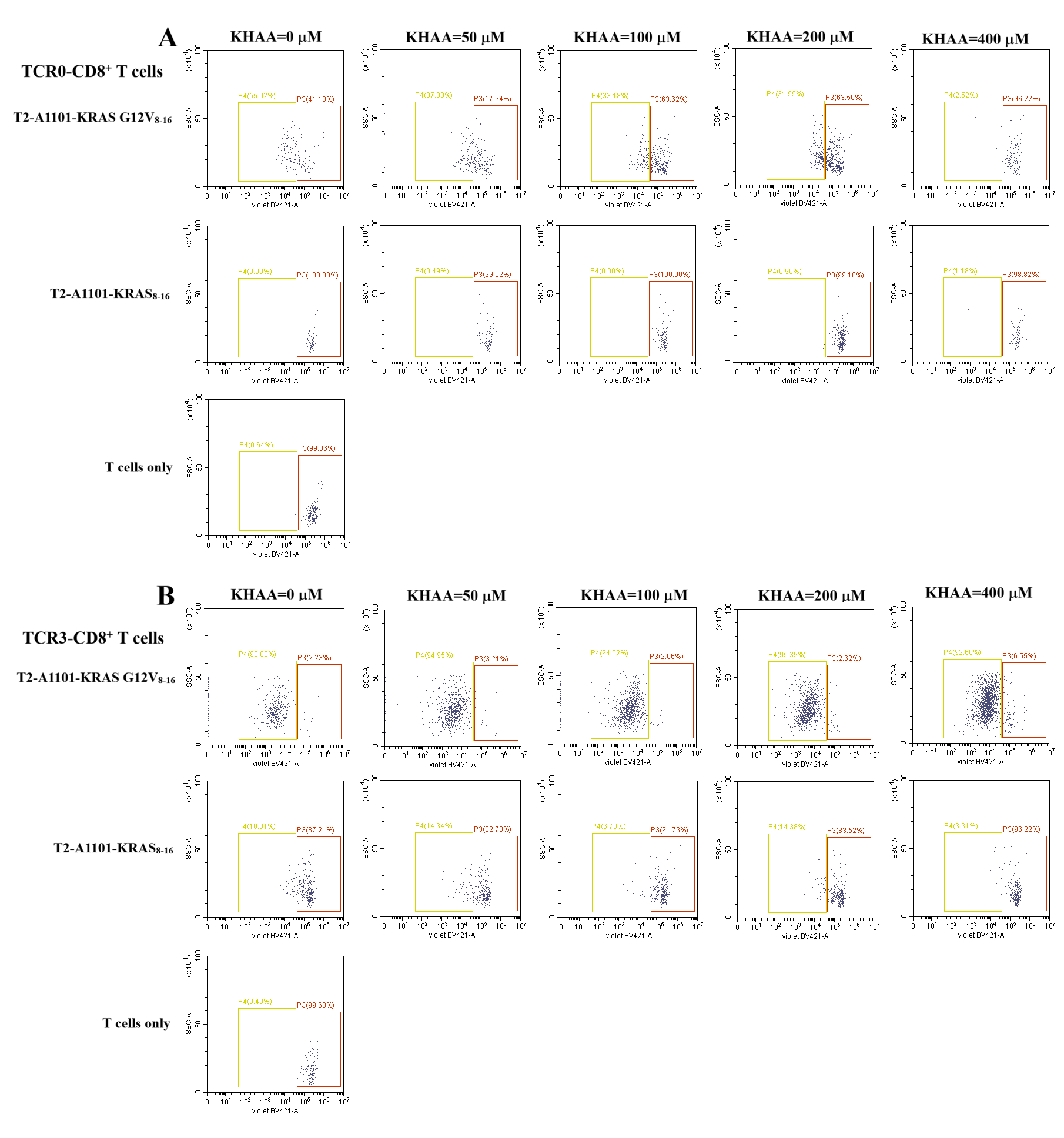


**Supplementary Figure 11. The representative FACS data exhibited the proportion of diminished CellTrace™ Violet from TCR-T cells**

After co-cultured with peptide (5E-6M)-pulsed T2-A1101 cells, the proliferation of TCR0-CD8^+^ T cells **(A)** and TCR3-CD8^+^ T cells **(B)** in the absence or presence of KHAA. TCR0-CD8^+^ T cells and TCR3-CD8^+^ T cells were pre-stained by CellTrace™ Violet, then respectively co-cultured with KRAS G12V_8-16_-peptide (5E-6M)-pulsed T2-A1101 cells or KRAS_8-16_-peptide (5E-6M)-pulsed T2-A1101 cells at an E:T ratio of 1:10 for 5 days. Cells were stained with FITC anti-mouse TCR β chain (1:100). The diminished CellTrace™ Violet from TCR-T cells was tested by flow cytometry (CytoFLEX S, Beckman Coulter) and represented the expanded T cells.


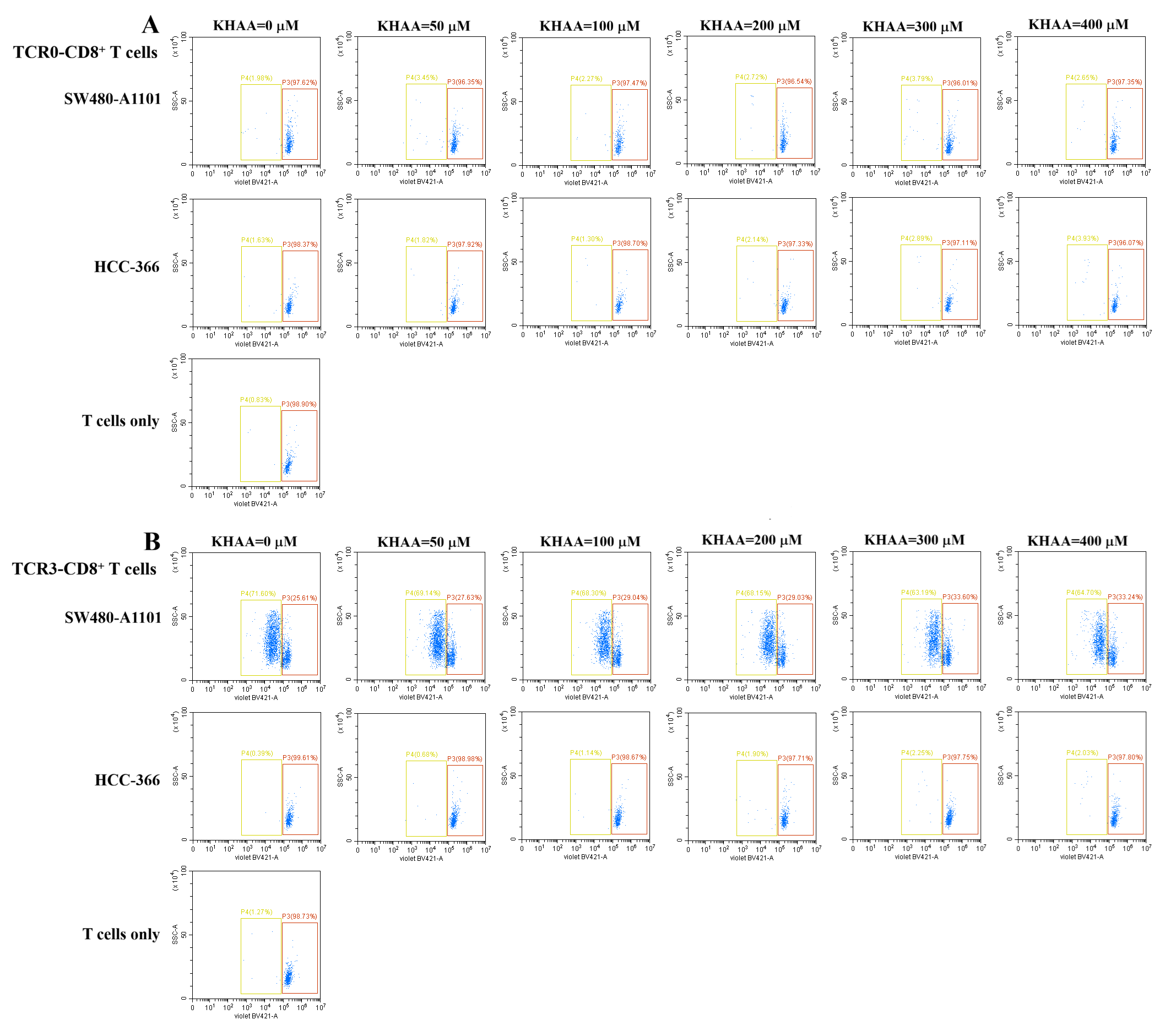


**Supplementary Figure 12. The representative FACS data exhibited the proportion of diminished CellTrace™ Violet from TCR-T cells**

After co-cultured with SW480-A1101 cells or HCC-366 cells, the proliferation of TCR0-CD8^+^ T cells **(A)** and TCR3-CD8^+^ T cells **(B)** in the absence or presence of KHAA. TCR0-CD8^+^ T cells and TCR3-CD8^+^ T cells were pre-stained by CellTrace™ Violet, then respectively co-cultured with SW480-A1101 cells or HCC-366 cells at an E:T ratio of 1:6 for 5 days. Cells were stained with FITC anti-mouse TCR β chain (1:100). The diminished CellTrace™ Violet from TCR-T cells were tested by flow cytometry (CytoFLEX S, Beckman Coulter) and represented the expanded T cells.


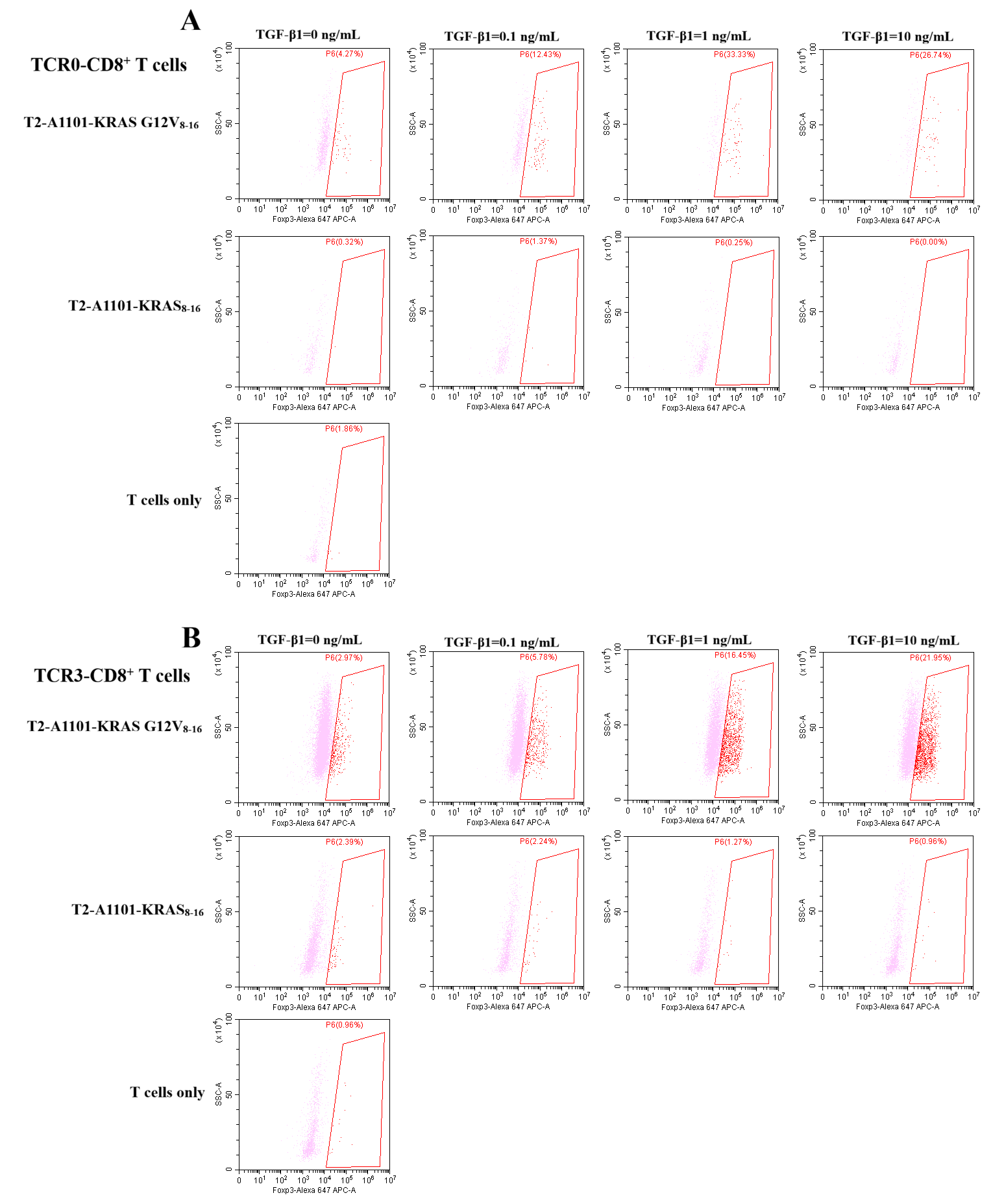


**Supplementary Figure 13. The representative FACS data exhibited the proportion of Foxp3 positive TCR-T cells**

After co-cultured with peptide (5E-6M)-pulsed T2-A1101 cells, the Foxp3 expression of TCR0-CD8^+^ T cells **(A)** and TCR3-CD8^+^ T cells **(B)** in the absence or presence of TGF‑β1. TCR0-CD8^+^ T cells and TCR3-CD8^+^ T cells were respectively co-cultured with KRAS G12V_8-16_-peptide (5E-6M)-pulsed T2-A1101 cells or KRAS_8-16_-peptide (5E-6M)-pulsed T2-A1101 cells at an E:T ratio of 1:10 for 5 days. Cells were firstly surface stained with PE anti-human CD8 Antibody (1:100) and FITC anti-mouse TCR β chain (1:100), then fixed and permeabilized with True-Nuclear™ Transcription Factor Buffer Set (Biolegend, Cat# 424401), following intracellular stained with Alexa Fluor^®^ 647 anti-human FOXP3 Antibody (1:25). The Foxp3 expression rates from TCR-T cells, were analyzed by ﬂow cytometer (CytoFLEX S, Beckman Coulter).


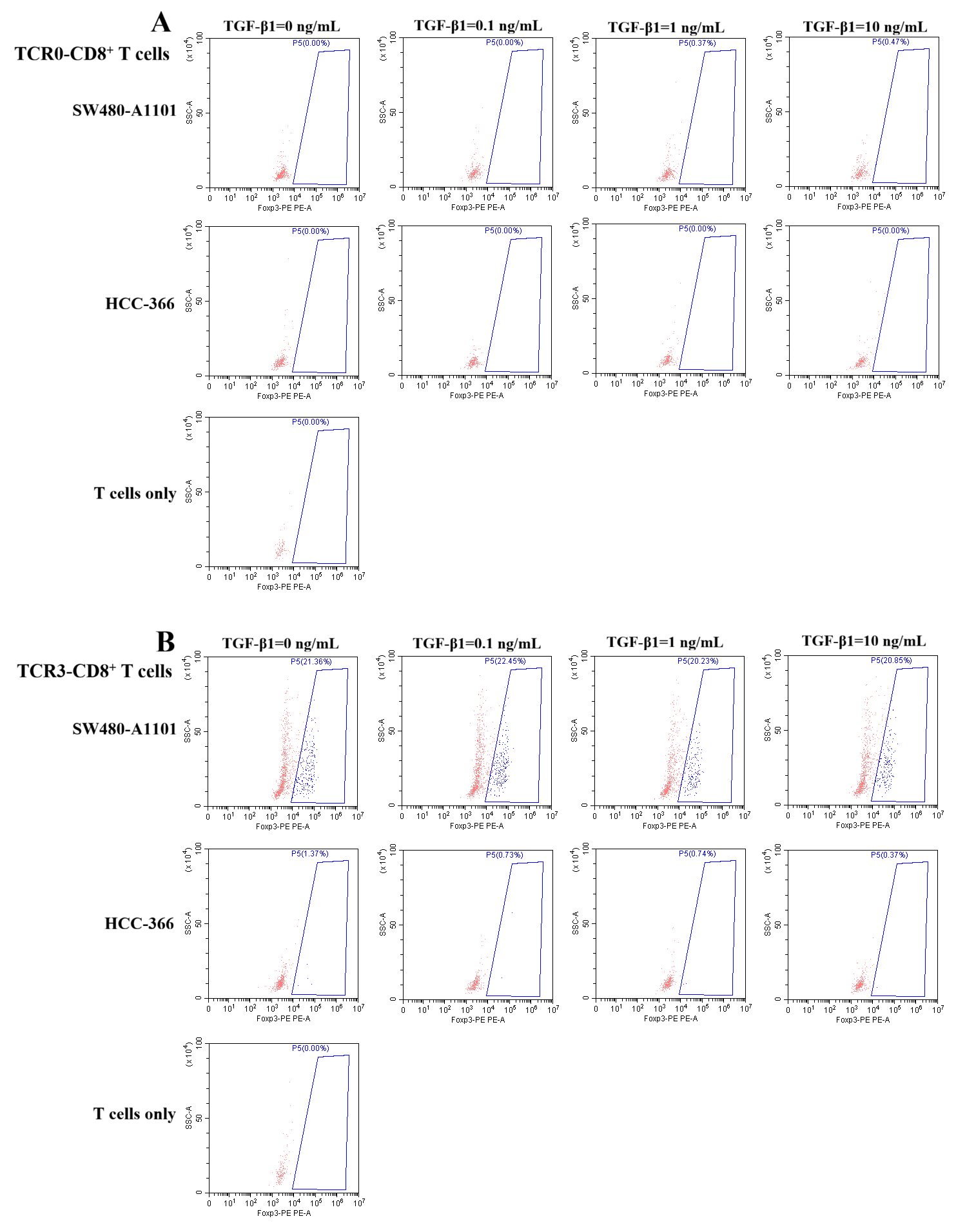


**Supplementary Figure 14. The representative FACS data exhibited the proportion of Foxp3 positive TCR-T cells**

After co-cultured with SW480-A1101 cells or HCC-366 cells, the Foxp3 expression of TCR0-CD8^+^ T cells **(A)** and TCR3-CD8^+^ T cells **(B)** in the absence or presence of TGF‑β1. TCR0-CD8^+^ T cells and TCR3-CD8^+^ T cells were respectively co-cultured with SW480-A1101 cells or HCC-366 cells at an E:T ratio of 1:5 for 5 days. Cells were firstly surface stained with APC anti-human CD8 Antibody (1:100) and FITC anti-mouse TCR β chain (1:100), then fixed and permeabilized with True-Nuclear™ Transcription Factor Buffer Set, following intracellular stained with PE anti-human FOXP3 Antibody (1:25). The Foxp3 expression rates from TCR-T cells, were analyzed by ﬂow cytometer (CytoFLEX S, Beckman Coulter).


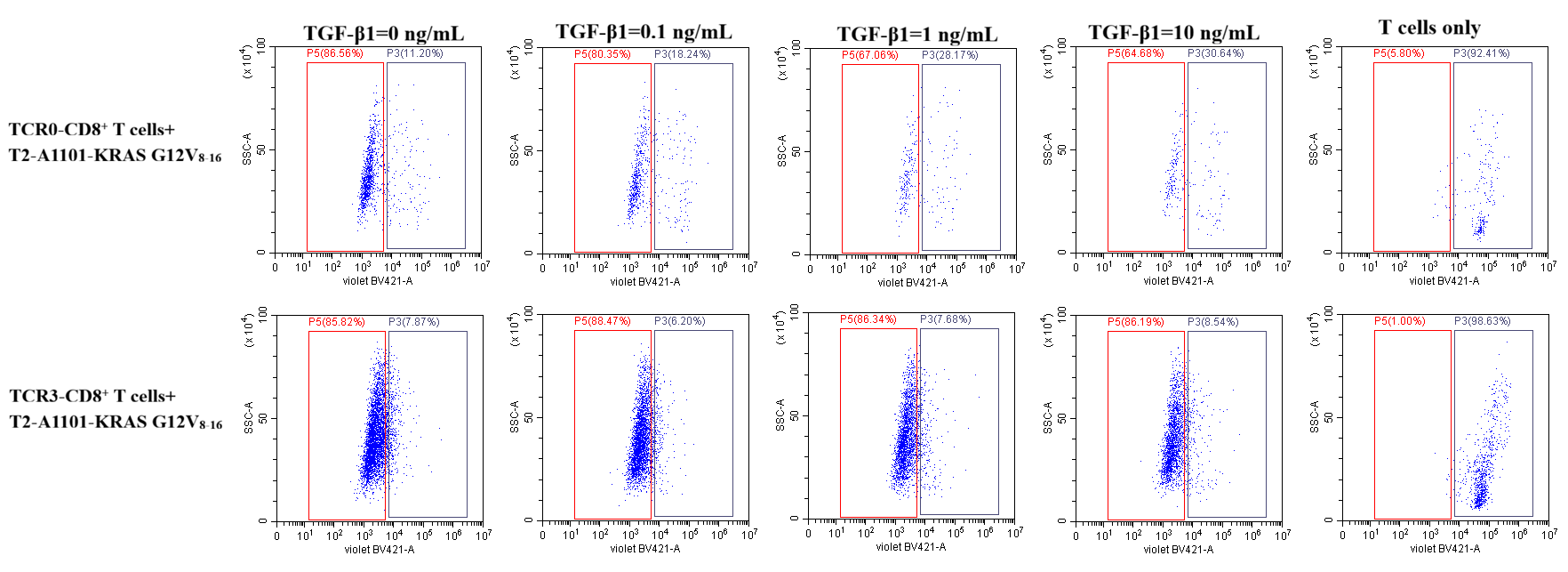


**Supplementary Figure 15. The representative FACS data exhibited the proportion of diminished CellTrace™ Violet from TCR-T cells**

TCR0-CD8^+^ T cells and TCR3-CD8^+^ T cells were pre-stained by CellTrace™ Violet, then respectively co-cultured with KRAS G12V_8-16_-peptide (5E-6M)-pulsed T2-A1101 cells at an E:T ratio of 1:10 for 5 days in the absence or presence of TGF‑β1. “T cells only” group was used as the background values. Cells were stained with PE anti-human CD8 Antibody (1:100) and FITC anti-mouse TCR β chain (1:100). The diminished CellTrace™ Violet from TCR-T cells were tested by flow cytometry (CytoFLEX S, Beckman Coulter) and represented the expanded T cells.


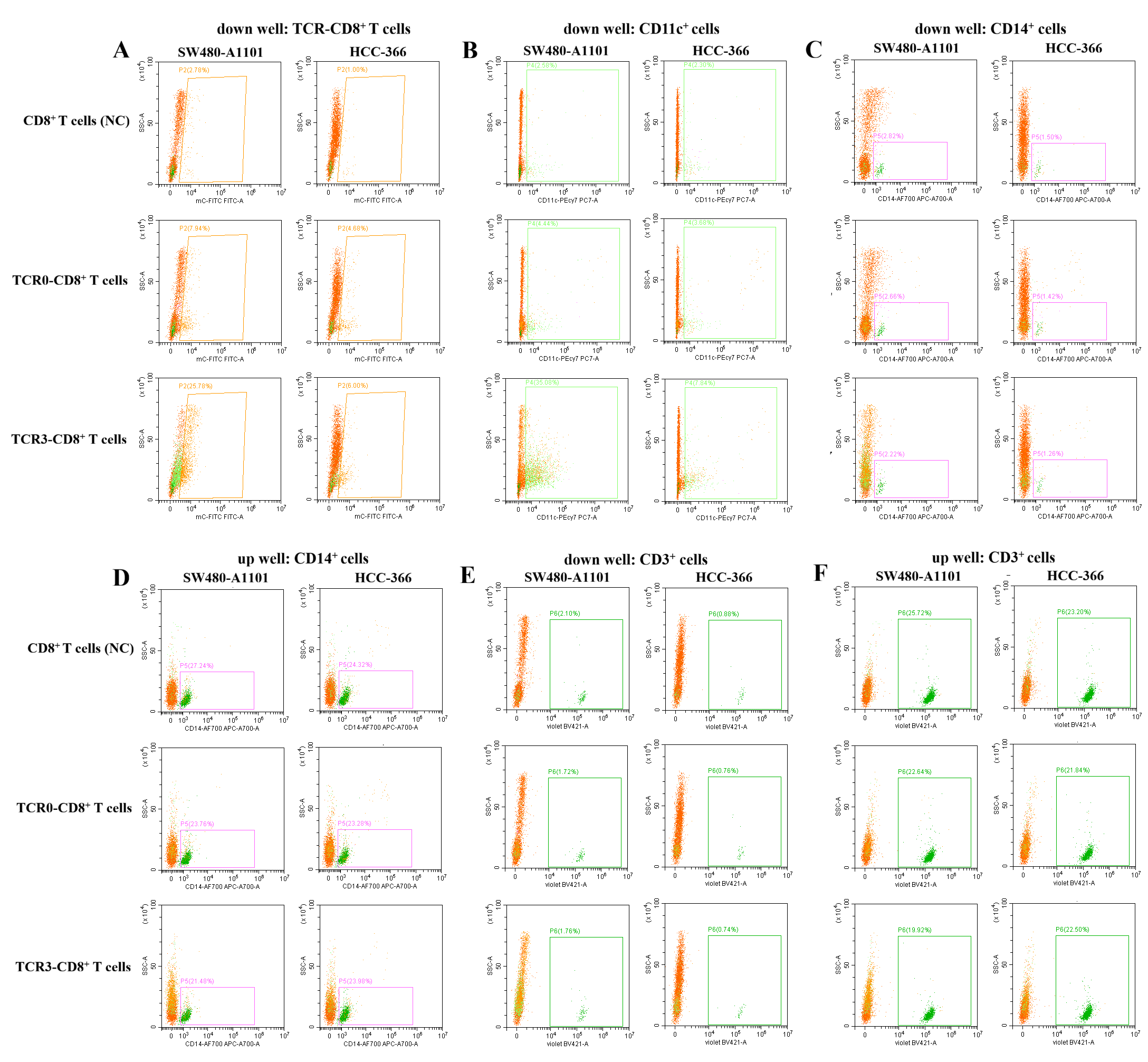


**Supplementary Figure 16. The representative FACS data exhibited the proportion of various subtype of immune cells**

**(A)** The proportion of TCR-T cells from down well of a transwell system. **(B)** The proportion of CD11c^+^ cells from down well of a transwell system. **(C)** The proportion of CD14^+^ cells from down well of a transwell system. **(D)** The proportion of CD14^+^ cells from up well of a transwell system. **(E)** The proportion of CD3^+^ cells from down well of a transwell system. **(F)** The proportion of CD3^+^ cells from up well of a transwell system. T-cell deleted PBMCs, TCR-T cells and CellTrace™ Violet pre-stained CD3^+^ cells were equally mixed to form mixed immune cells. Using an E:T ratio of 1:1, the target cells (SW480-A1101 cells or HCC-366 cells) were plated in the down well of a transwell system, and the mixed immune cells were plated in the up well of a transwell system. After 3-day co-culture, cells from down well or up well were collected and simultaneously stained with FITC anti-mouse TCR β chain (1:100), Alexa Fluor^®^ 700 anti-human CD14 (1:100), PE/Cyanine7 anti-human CD11c (1:100). The various subtype of immune cells, including TCR-T cells, CD11c^+^ cells, CD14^+^ cells and CD3^+^ cells, were analyzed by ﬂow cytometer (CytoFLEX S, Beckman Coulter).


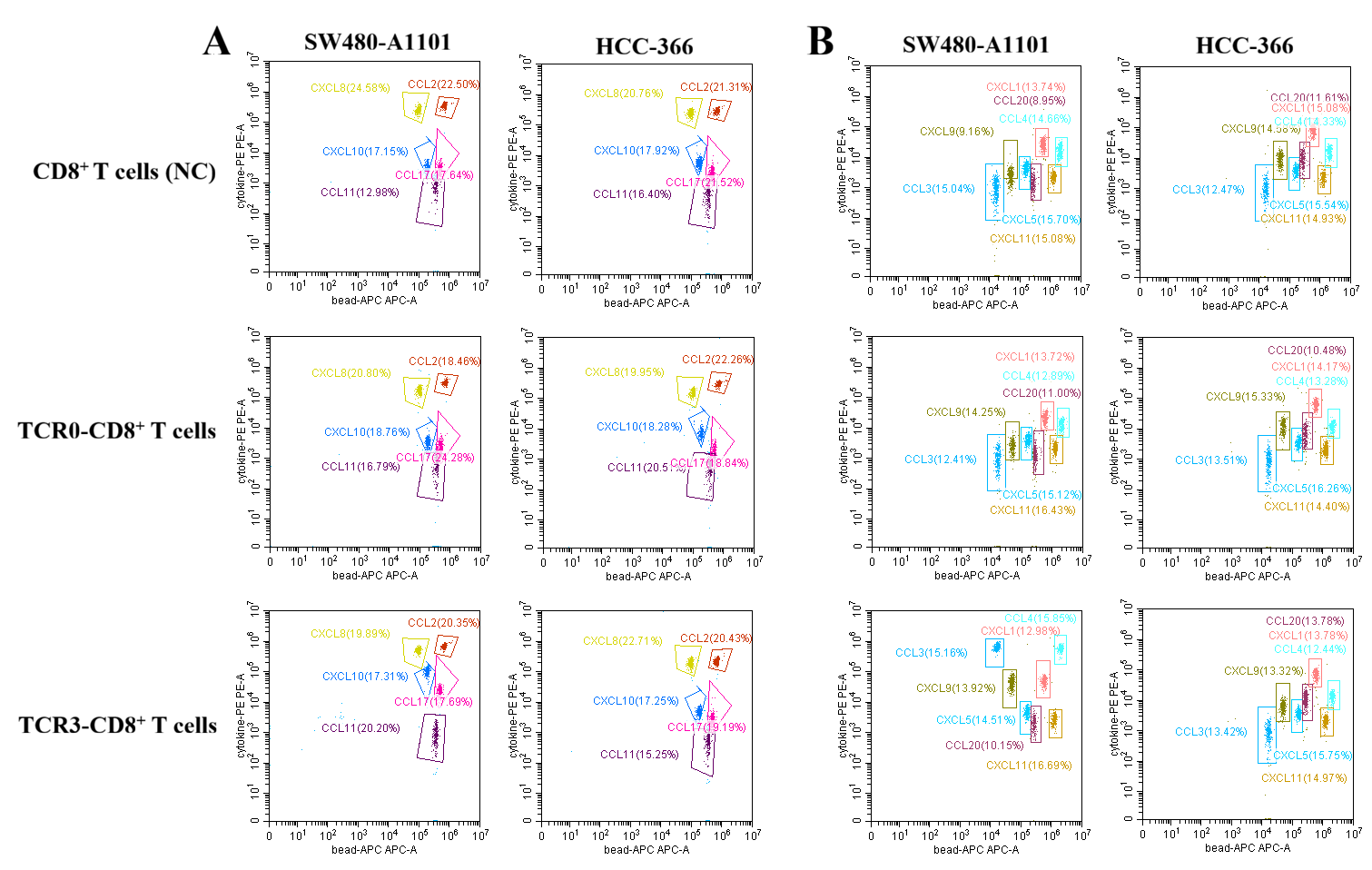


**Supplementary Figure 17. The representative FACS data exhibited the fluorescence intensity of chemokines from various groups**

**(A)** The fluorescence intensity of 5 kinds of chemokines. **(B)** The fluorescence intensity of 7 kinds of chemokines. T-cell deleted PBMCs, TCR-T cells and CellTrace™ Violet pre-stained CD3^+^ cells were equally mixed to form mixed immune cells. Using an E:T ratio of 1:1, the target cells (SW480-A1101 cells or HCC-366 cells) were plated in the down well of a transwell system, and the mixed immune cells were plated in the up well of a transwell system. After 3-day co-culture, the medium from down well were collected. According to the manufacturer’s instructions, 12 kinds of chemokines contained in the suspension were tested by LEGENDplex^TM^ HU Proinflam Chemokine Panel 1. The fluorescence signals of chemokines were analyzed through ﬂow cytometer (CytoFLEX S, Beckman Coulter).


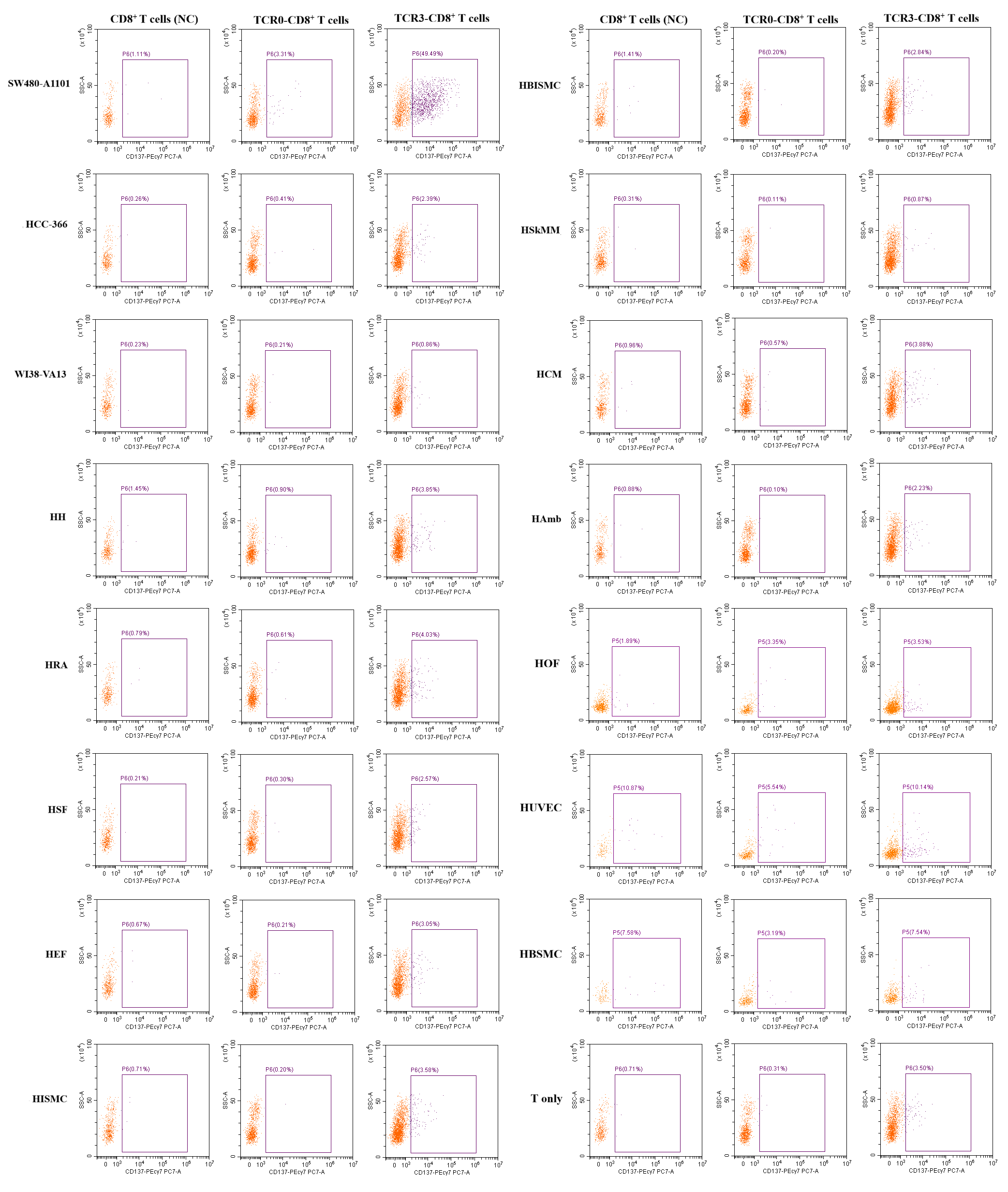


**Supplementary Figure 18. The representative FACS data exhibited the proportion of CD137 positive TCR-T cells**

TCR3-CD8^+^ T cells, TCR0-CD8^+^ T cells or CD8^+^ T cells were respectively co-cultured with SW480-A1101 cells or HCC-366 cells at an E:T ratio of 5:1 for 16 h. Cells were simultaneously stained with FITC anti-mouse TCR β chain (1:100) and PE/Cyanine7 anti-human CD137 (4-1BB) Antibody (1:100). The CD137 rates from TCR-T cells, which were DAPI negative, were analyzed by ﬂow cytometer (CytoFLEX S, Beckman Coulter).


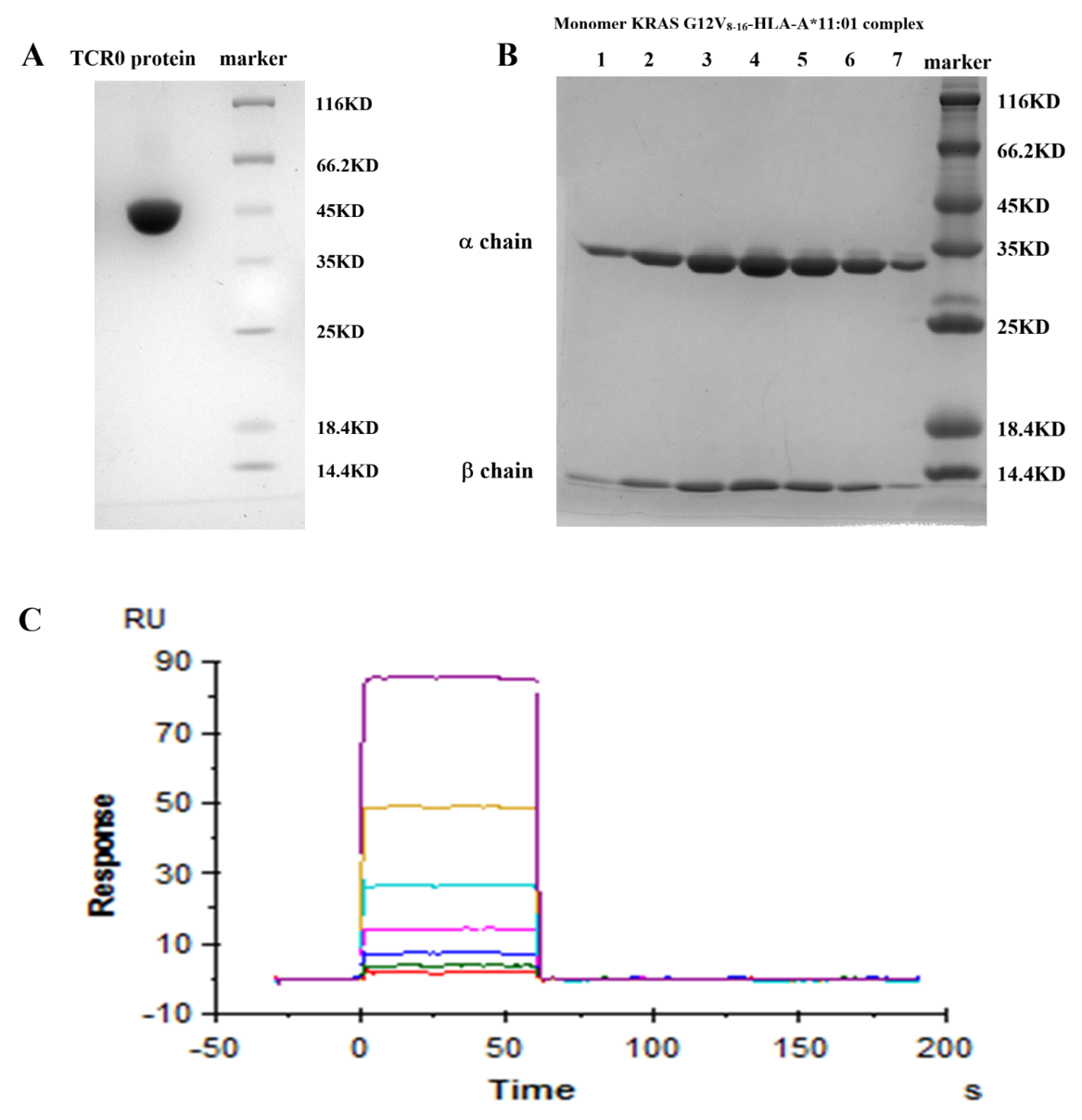


**Supplementary Figure 19. The affinity detection of TCR0 binding KRAS G12V_8-16_-HLA-A*11:01 complex**

**(A)** The preparation of TCR0 protein. The extracellular regions of TCR0 α and β chains were respectively expressed from *E.coli* as inclusion body. Inclusion bodies of TCR0 α and β chains were purified. After denaturing the inclusion bodies, the TCR0 protein was prepared by refolding with refolding buffer (5 mM urea, 100 mM Tris (pH 8.1), 0.4 mM L-arginine, 2 mM EDTA, 6.5 mM cysteamine, 1.87 mM cysteine) combined with dialysis. The dialyzed suspension containing refolded TCR0 was purified through anion exchange and chromatography. The purified TCR0 was detected on non-reducing SDS-PAGE. The purity of TCR0 was over 90%, and the molecular weight of TCR0 was consistent with its theoretical value. **(B)** The preparation of monomer KRAS G12V_8-16_-HLA-A*11:01 complex. The extracellular regions of HLA-A*11:01 α and β chains were respectively expressed from *E.coli* as inclusion body. Inclusion bodies of HLA-A*11:01 α and β chains were purified, mixed with KRAS G12V_8-16_ peptide, then refolded through refolding buffer combined with dialysis. The dialyzed suspension containing refolded monomer KRAS G12V_8-16_-HLA-A*11:01 complex was purified through anion exchange and chromatography. The purified monomer KRAS G12V_8-16_-HLA-A*11:01 complex was detected on reducing SDS-PAGE. The purity of monomer KRAS G12V_8-16_-HLA-A*11:01 complex was over 90%, and the molecular weights of α and β chains were consistent with their theoretical values. Lane 1 to 7 represented the different wells from chromatography samples. **(C)** The affinity of TCR0 detected by Surface Plasmon Resonance (SPR). The CM5 chip was pre-coated with streptavidin, and the biotinylated KRAS G12V_8-16_-HLA-A*11:01 complex was flowed through as the detection channel, while the other channel served as the reference channel. The remaining binding sites of streptavidin were blocked with biotin. The refolded TCR0 protein was diluted to different concentrations (such as 1 μM, 2 μM, 4 μM, 8 μM, 16 μM, 32 μM, and 64μM), and sequentially flowed over the chip surface at a flow rate of 30 μL/min. Each injection had a binding time (such as 60 seconds), followed by a dissociation period (such as 130 seconds) after the final injection. The kinetic parameters were calculated using the BIAcore Evaluation software (Supplementary Table 6).


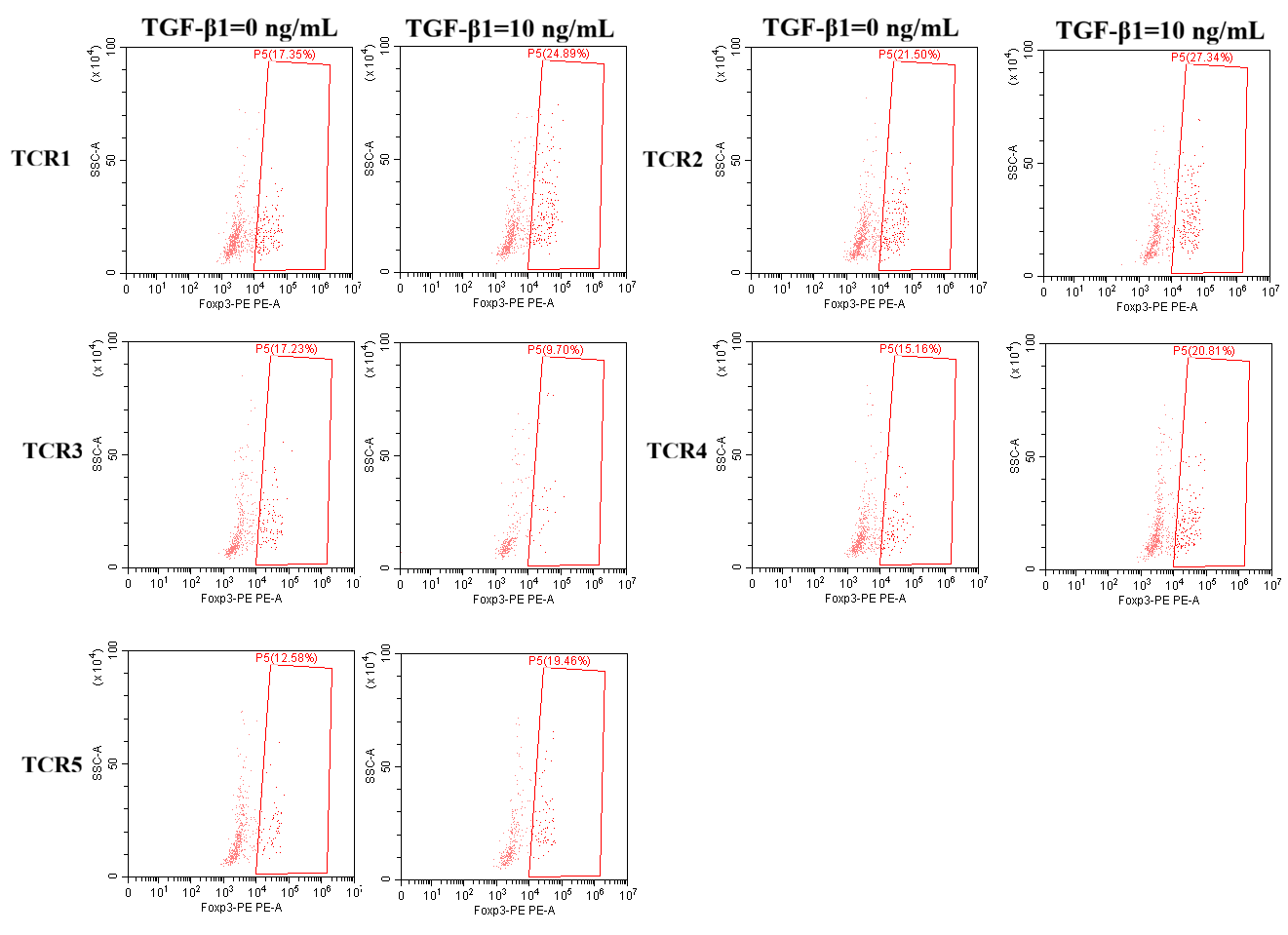


**Supplementary Figure 20. The representative FACS data exhibited the proportion of Foxp3 positive TCR-T cells**

In the presence or absence of TGF-β1, TCR1-CD8^+^ T cells, TCR2-CD8^+^ T cells, TCR3-CD8^+^ T cells, TCR4-CD8^+^ T cells, or TCR5-CD8^+^ T cells were respectively co-cultured with SW480-A1101 cells at an E:T ratio of 1:5 for 5 days. Cells were firstly surface stained with APC anti-human CD8 Antibody (1:100) and FITC anti-mouse TCR β chain (1:100), then fixed and permeabilized with True-Nuclear™ Transcription Factor Buffer Set, following intracellular stained with PE anti-human FOXP3 Antibody (1:25). The Foxp3 expression rates from TCR-T cells, were analyzed by ﬂow cytometer (CytoFLEX S, Beckman Coulter).
